# Supplementary material for: Non-canonical Phototransduction Mediates Synchronization of the Drosophila melanogaster Circadian Clock and Retinal Light Responses
Source: Curr Biol. 2018 Jun 4;28(11):1725–1735.e3. doi: 10.1016/j.cub.2018.04.016 (PMC5988559; doi:10.1016/j.cub.2018.04.016)
Supplement: Document S1. Figures S1–S3 and Table S1 [file mmc1.pdf]

Current Biology, Volume *28*

Supplemental Information

Non-canonical Phototransduction Mediates Synchronization of the *Drosophila melanogaster* Circadian Clock and Retinal Light Responses

Maite Ogueta, Roger C. Hardie, and Ralf Stanewsky

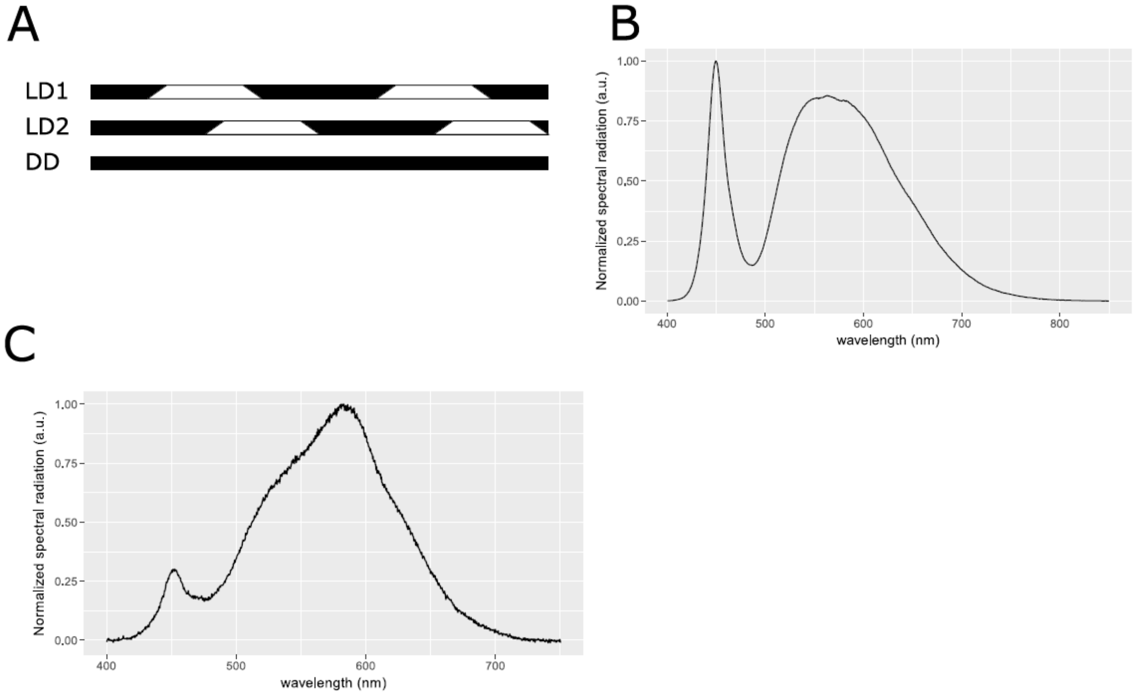

Figure S1. Characteristics of light conditions used in the experiments. **Related to Figures 1, 2, 4, 5.** (A) Light profile during the behavioural tests. During both LD periods, the lights are ramped for two hours simulating dawn and dusk respectively. The phase of LD1 is the same one as where the flies where reared, while LD2 has a phase shift of 6 hours. (B) Spectrum of the cold white LEDs used in the experiment. (C) Spectrum of the light source used for ERG recordings.

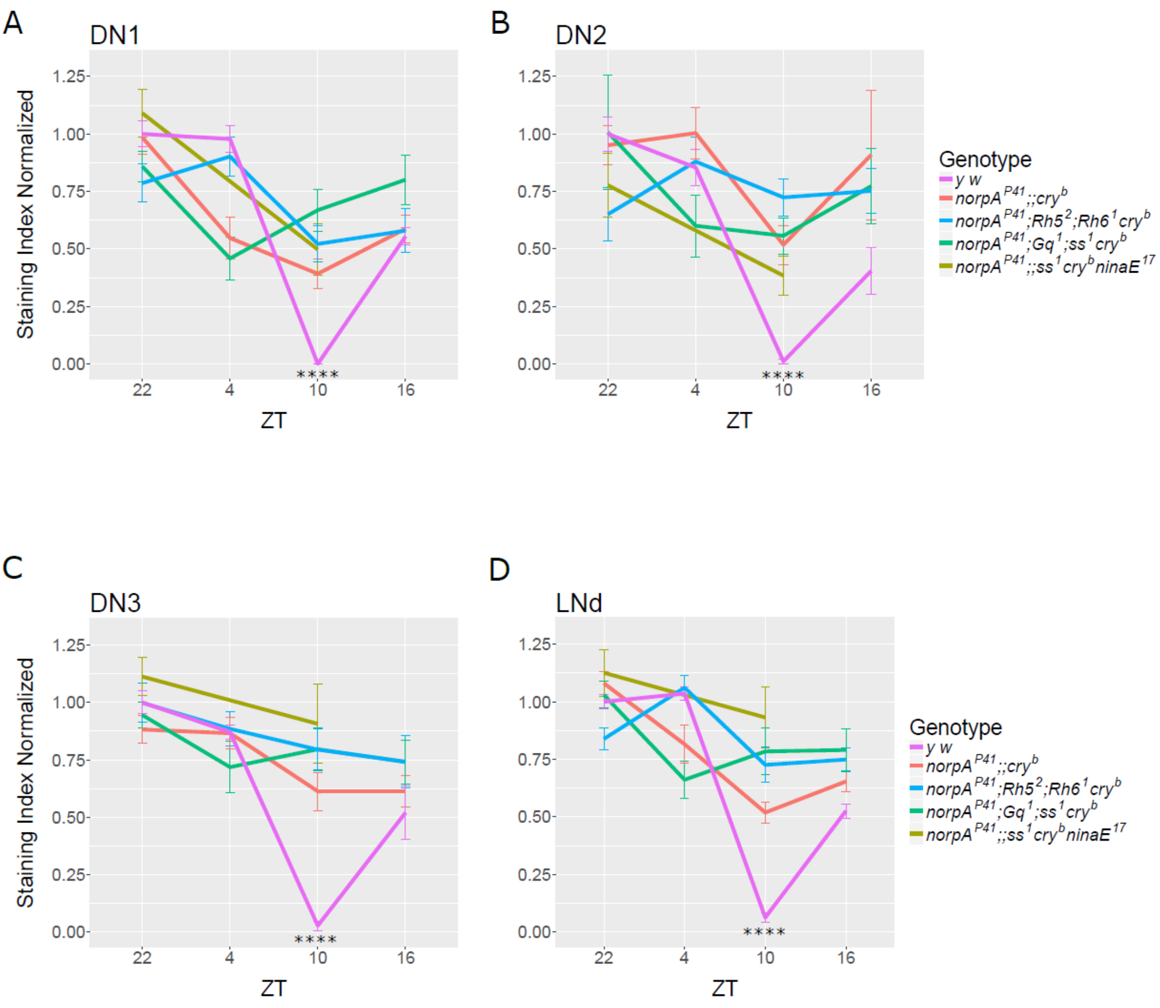

Figure S2. PER does not oscillate in the DNs and LNds of *norpA*^{P41};;*cry*^{b} flies. Related to Figure 3. Quantification of PER expression in DN1 (A), DN2 (B), DN3 (C) and LNd (D) of the different genotypes, normalized to the values of *y w* at ZT22.Only *y w* flies show a trough of PER expression at ZT10 for all neuronal groups. Error bars represent SEM (n numbers in Table S1). **** represents p<0.0000001.

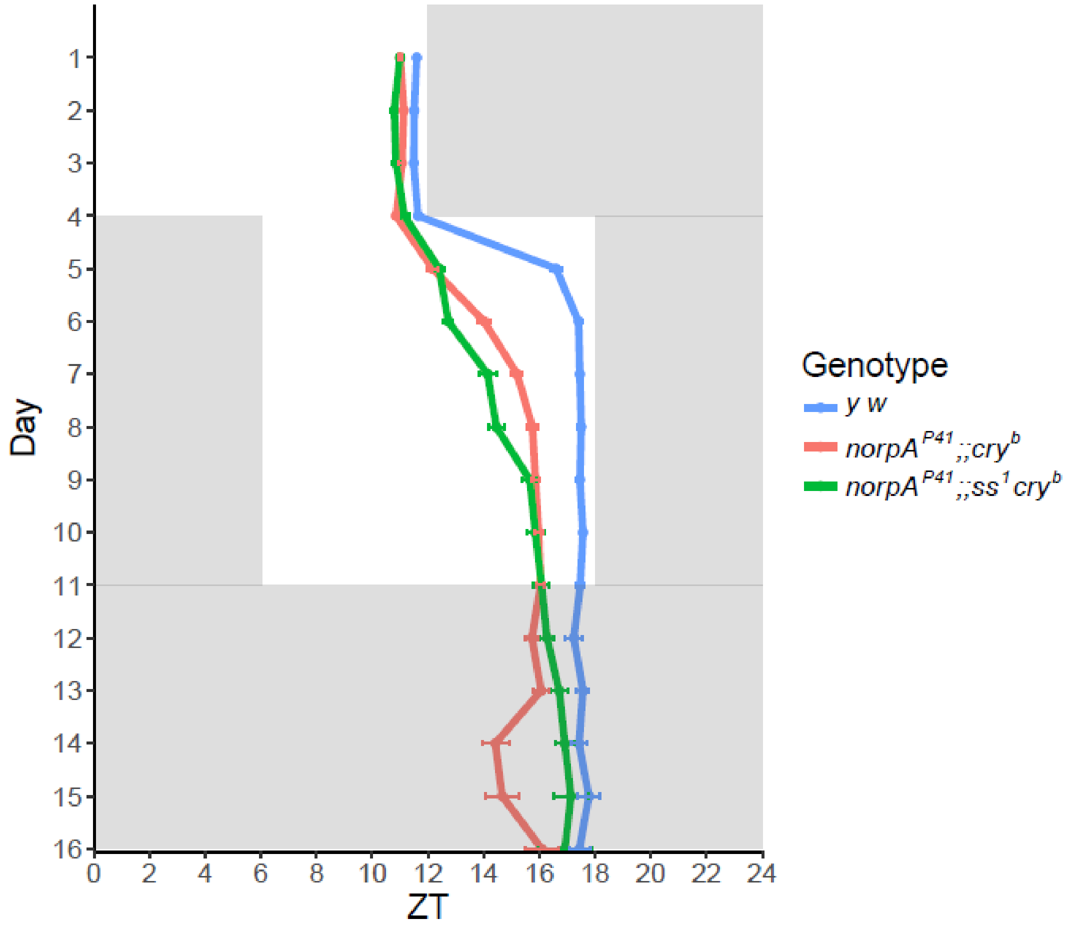

Figure S3. The hypomorphic *ss*^{1} allele does not further compromise resynchronisation of *norpA*^{P41} *cry*^{b} flies. Related to Figure 2. Flies of the indicated genotypes were tested for resynchronisation to a 6 hr delayed LD cycle as in Figures 1, 2, and 5. Because loss-of-function alleles of *ss* reduce expression of Rh6 in R8 cells [S1], we tested if *norpA*^{P41} *ss*^{1} *cry*^{b} flies needed longer for resynchronisation compared to *norpA*^{P41} *cry*^{b} flies. There was no significant differences between both genotypes, demonstrating that the lack of resynchronisation in *norpA*^{P41} *ninaE*^{17} *ss*^{1} *cry*^{b} flies (Figure 2C) is caused by the absence of Rh1 and not by reduction in Rh6 (see Discussion for details). n: *y w* 58, *norpA*^{P41} *cry*^{b} 40, *norpA*^{P41} *ss*^{1} *cry*^{b} 21.

Table S1. Number of hemispheres quantified for PER expression. Related to Figure 2 and S2.

Genotype	ZT	Hemispheres
y w	22	52
	4	8
	10	37
	16	8
norpA^{P41};;cry^{b}	22	51
	4	19
	10	24
	16	17
norpA^{P41};Rh5^{2};Rh6^{1} cry^{b}	22	18
	4	7
	10	23
	16	11
norpA^{P41};Gq^{1}; ss^{1}cry^{b}	22	35
	4	12
	10	21
	16	6
norpA^{P41};;ninaE^{17} ss^{1} cry^{b}	22	13
	10	12

Numbers correspond to Zeitgeber Time (ZT) during a 12 hr : 12 hr LD cycle at 25°C (lights on at ZT0, lights off at ZT12) and to the number of hemispheres that were quantified at a given ZT for each genotype. Brains were sampled from 2 to 5 independent experiments.

Supplemental References

S1. Wernet, M.F., Mazzoni, E.O., Çelik, A., Duncan, D.M., Duncan, I., and Desplan, C. (2006). Stochastic spineless expression creates the retinal mosaic for colour vision. Nature *440*, 174–180.
