# Supplementary material for: Non-canonical Phototransduction Mediates Synchronization of the Drosophila melanogaster Circadian Clock and Retinal Light Responses
Source: Curr Biol. 2018 Jun 4;28(11):1725–1735.e3. doi: 10.1016/j.cub.2018.04.016 (PMC5988559; doi:10.1016/j.cub.2018.04.016)
Supplement: Document S2. Article plus Supplemental Information [file mmc2.pdf]

Current Biology

Article

Non-canonical Phototransduction Mediates Synchronization of the *Drosophila melanogaster* Circadian Clock and Retinal Light Responses

Graphical Abstract

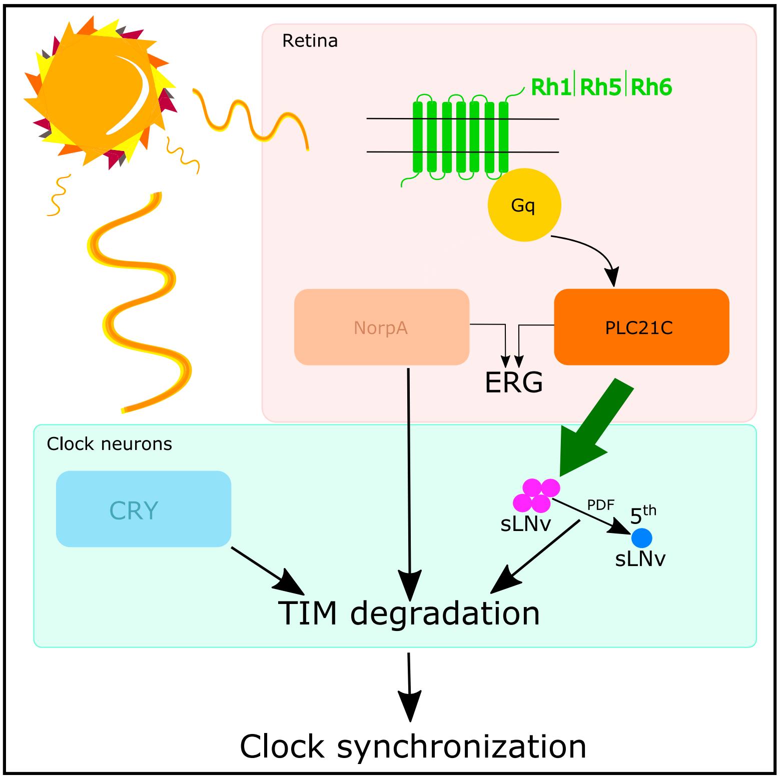

Authors

Maite Ogueta, Roger C. Hardie, Ralf Stanewsky

Correspondence

stanewsky@uni-muenster.de

In Brief

Light synchronization of the *Drosophila* circadian clock relies on cryptochrome and rhodopsin photoreceptors. Ogueta et al. reveal that rhodopsin signaling to the clock involves non-canonical phototransduction, which synchronizes key circadian pacemaker neurons and behavior. In the eye, the same pathway can mediate ERG responses to brief light pulses.

Highlights

- CRYPTOCHROME-independent light input synchronizes key circadian pacemaker neurons
- In the absence of *norpA, Plc21C* mediates this novel light input
- Rhodopsin 1, Rhodopsin 5, and Rhodopsin 6 can signal via this Gq-dependent pathway
- The same pathway mediates ERG responses from R8 photoreceptors in *norpA* mutants

Ogueta et al., 2018, Current Biology *28*, 1725–1735
June 4, 2018 © 2018 The Authors. Published by Elsevier Ltd.
https://doi.org/10.1016/j.cub.2018.04.016

CellPress

Current Biology
Article

CellPress

Non-canonical Phototransduction Mediates Synchronization of the *Drosophila melanogaster* Circadian Clock and Retinal Light Responses

Maite Ogueta,[1] Roger C. Hardie,[2] and Ralf Stanewsky[1,3,*]
[1]Institute of Neuro- and Behavioral Biology, Westfälische Wilhelms University, 48149 Münster, Germany
[2]Department of Physiology, Development, and Neuroscience, Cambridge University, Cambridge CB2 3DY, UK
[3]Lead Contact
*Correspondence: stanewsky@uni-muenster.de
https://doi.org/10.1016/j.cub.2018.04.016

SUMMARY

The daily light-dark cycles represent a key signal for synchronizing circadian clocks. Both insects and mammals possess dedicated "circadian" photoreceptors but also utilize the visual system for clock resetting. In *Drosophila*, circadian clock resetting is achieved by the blue-light photoreceptor cryptochrome (CRY), which is expressed within subsets of the brain clock neurons. In addition, rhodopsin-expressing photoreceptor cells contribute to light synchronization. Light resets the molecular clock by CRY-dependent degradation of the clock protein Timeless (TIM), although in specific subsets of key circadian pacemaker neurons, including the small ventral lateral neurons (s-LNvs), TIM and Period (PER) oscillations can be synchronized by light independent of CRY and canonical visual Rhodopsin phototransduction. Here, we show that at least three of the seven *Drosophila* rhodopsins can utilize an alternative transduction mechanism involving the same α-subunit of the heterotrimeric G protein operating in canonical visual phototransduction (Gq). Surprisingly, in mutants lacking the canonical phospholipase C-β (PLC-β) encoded by the *no receptor potential A* (*norpA*) gene, we uncovered a novel transduction pathway using a different PLC-β encoded by the *Plc21C* gene. This novel pathway is important for behavioral clock resetting to semi-natural light-dark cycles and mediates light-dependent molecular synchronization within the s-LNv clock neurons. The same pathway appears to be responsible for *norpA*-independent light responses in the compound eye. We show that Rhodopsin 5 (Rh5) and Rh6, present in the R8 subset of retinal photoreceptor cells, drive both the long-term circadian and rapid light responses in the eye.

INTRODUCTION

Circadian clocks regulate the physiology, behavior, and sleep of organisms in a rhythmic daily fashion. Proper timing of these parameters contributes to overall fitness, and it is, therefore, crucial that circadian clocks are accurately synchronized with the environmental time, dictated by the natural daily cycles of light and temperature [1]. Absence of any environmental time cues reveals the endogenous nature of circadian clock function, realized by rhythmic expression of clock genes. In *Drosophila*, the key clock genes *period* and *timeless* are rhythmically activated by CLOCK (CLK) and CYCLE (CYC), transcription factors belonging to the basic-helix-loop-helix (bHLH)/PAS domain family [2]. Period (PER) and Timeless (TIM) proteins accumulate in the cytoplasm, whereby PER is stabilized by dimerization with TIM. Eventually, PER and TIM translocate to the nucleus and block CLK/CYC activity to shut down their own transcription until eventual degradation of PER and TIM restarts the cycle [2]. These molecular oscillations can be synchronized to the environment by light-induced degradation of TIM, which is mediated by the blue light photoreceptor cryptochrome (CRY) [3, 4]. Upon light exposure, CRY undergoes a conformational change, allowing it to bind to TIM and the F-box protein JETLAG, resulting in the proteasomal degradation of TIM and CRY [5–10]. Because PER stability depends on the presence and binding to TIM, this pathway efficiently resets the circadian clock to light.

Although the circadian clock mechanism operates in many cells throughout the body, only a few neurons in the fly brain (~150) express clock genes. These interconnected "clock neurons" form a neuronal network that regulates the fly's daily behavioral activity rhythm, representing the best studied output rhythm controlled by the clock [11]. Based on the position within the brain and expression of the neuropeptide Pigment Dispersing Factor (PDF), clock neurons are subdivided into PDF-expressing small and large ventral lateral neurons (s-LNvs and l-LNvs, respectively), the fifth PDF-negative s-LNv, the dorsal lateral neurons (LNds), the dorsal neuron groups 1, 2, and 3 (DN1, DN2, and DN3), and the lateral posterior neurons (LPNs) [11]. The l-LNvs are important cells for the regulation of sleep and arousal behavior and are thought to propagate light input to the clock [12–15]. The s-LNvs are the key neurons driving behavioral rhythms in constant darkness [16] but also regulate anticipatory behavioral activity at dawn, which is why they were also coined Morning (M) cells [17, 18]. The fifth s-LNv and LNd represent the Evening (E) cells, which are important for anticipatory behavioral activity at dusk. The DNs form heterogeneous groups of neurons that receive circadian signals from the other clock neurons but also receive and deliver light and temperature information to the network [19].

This is an open access article under the CC BY license (http://creativecommons.org/licenses/by/4.0/).

CRY is expressed in about 50% of the central brain clock neurons, including all of the LNvs, and subsets of the LNd and DN1. Apart from CRY, circadian clock resetting of behavioral activity and the underlying molecular rhythms in clock neurons is mediated by the compound eyes, the Hofbauer-Buchner eyelet (H-B eyelet), and the ocelli, e.g [4, 20–23]. At least six different rhodopsins are expressed in the various visual photoreceptors. The major Rhodopsin 1 (Rh1) is present in the outer photoreceptors, while Rh3–Rh6 are expressed in the inner photoreceptors (Rh3 and Rh4 in R7 and Rh5/6 in R8). Rh6 is also the opsin expressed in the H-B eyelet, while the ocelli only contain Rh2 [24, 25]. The expression pattern of the recently characterized Rh7 is less clear, as different studies report expression in the s-LNv and LNd [26], or within the subset of the DN1, and the R8 and H-B-eyelet photoreceptors [27], respectively.

Flies lacking CRY can still synchronize their circadian clock to light-dark (LD) cycles, and the same applies for visually blind flies lacking *norpA*-encoded phospholipase C-β (PLC-β), an essential enzyme in the canonical phototransduction cascade operating in the visual photoreceptor cells [4]. When visual phototransduction is blocked in parallel with removing CRY (e.g., in *norpA^{P41} cry^{b}* double mutants), flies lose the ability to synchronize to low light intensities (5–16 lux); nevertheless, they remain able to slowly synchronize to higher light intensities (400 to 1,000 lux) [4, 22, 28, 29]. In line with these behavioral observations, s-LNvs, LNds, and DN1s exhibit light-synchronized clock protein oscillations in the absence of CRY [4, 20, 30–32]. Moreover, s-LNvs of *norpA^{P41} cry^{b}* mutants can still be synchronized by LD cycles, suggesting that the LNds receive *norpA*-dependent, and the s-LNvs receive *norpA*-independent, visual system light input [20, 31]. Szular et al. (2012) showed that Rh5 and Rh6 contribute to *norpA*-independent behavioral synchronization, raising the exciting possibility that these rhodopsins can use alternative phototransduction pathways and that they are responsible for the CRY-independent light synchronization of TIM and PER expression in the s-LNvs [20, 28].

Here, we tested this hypothesis by combining different rhodopsin and phototransduction mutants with the *norpA^{P41} cry^{b}* double mutant. We show that *norpA*- and *cry*-independent light input specifically targets the s-LNvs by mediating light-dependent degradation of clock proteins within these neurons. *norpA*- and *cry*- independent molecular and behavioral synchronization depends on Rh1, Rh5, and Rh6 and also requires Gq as well as the second *Drosophila* PLC-β, encoded by *Plc21C*. Electroretinogram (ERG) recordings from the same mutants reveal residual *norpA*-independent light responses that depend on *Rh5* and *Rh6* expressed in R8 photoreceptors as well as on *Gq* and *Plc21C*.

RESULTS

norpA-Independent Circadian Clock Resetting Requires Gq

As a measure of circadian clock synchronization to light, wild-type and mutant flies were raised in 12-hr:12-hr LD cycles (lights on at 9 a.m.) at a constant temperature of 25°C and 60% relative humidity. During the light part of these LD cycles, 3- to 5-day-old males were individually loaded into glass tubes, and locomotor activity was monitored for 4 days using the same LD cycle (i.e., lights on at 9 a.m.), except that now, light intensity was gradually ramped from 0 to 180 lux and back, using white LEDs (see Figure S1 for light profile and LED spectrum). We avoided using higher light intensities to prevent potential light-independent activation of rhodopsins [33]). To ensure that all mutant (potentially "circadian blind") genotypes become synchronized during the first 4 days of the experiment, we applied a 25°C:16°C temperature cycle combined with the LD cycle (temperature increase at 9 a.m.). During these conditions, wild-type flies exhibit typical bimodal or crepuscular behavior, with activity peaks in the morning and evening (Figure 1A) [34]. After 4 days of exposure to these combined LD and temperature cycles, a clear synchronized activity peak in all genotypes could be observed (Figures 1 and 2). In contrast to the wild-type controls, most of the visually impaired mutants exhibited a slowly advancing or broadened evening activity peak, which is reminiscent of the wild-type activity peak after exposure to 25°C:16°C temperature cycles in constant darkness (DD) (e.g., Figures 2A–2D) [34]. Although this preferential synchronization to temperature already indicates deficits in light resetting, the synchronized activity peak allowed us in the following to analyze how many days the various genotypes require to resynchronize their behavioral activity pattern to a 6-hr delay of this LD cycle (where the temperature was kept constant at 25°C). Flies were exposed for 7 days to the shifted LD cycle (mimicking a rapid westward time zone shift of 6 hr) before being released into DD conditions at constant 25°C. As previously reported, wild-type flies and *norpA^{P41}* mutants require only 1–2 days for resynchronization, whereas *cry^{b}* and *norpA^{P41} cry^{b}* double mutants require at least 5 days [28, 29] (Figures 1A–1D, 2A, and 2E). Additional removal of Rh5 and Rh6 function (*norpA^{P41} Rh5^{2} Rh6^{1} cry^{b}*) abolishes the ability to resynchronize completely, confirming that Rh5 and Rh6 operate in a *norpA*- and *cry*-independent pathway (Figures 2B and 2E) [28]. We then tested flies lacking Rh1 (encoded by *ninaE*) in the same *norpA^{P41} cry^{b}* background and found that they, too, are not able to resynchronize to LD cycles, suggesting that Rh1 can also signal independently of *norpA* (Figure 2C). In order to identify where the *norpA*-dependent and *norpA*-independent transduction pathways diverge, we analyzed *Gq^{1}* mutant flies in the background of the *norpA^{P41} cry^{b}* double mutant. These mutants also failed to resynchronize to the shifted LD cycle, indicating that the signaling pathways diverge after activation of Gq and before activation of the *norpA*-encoded PLC-β (Figure 2D). We note that removal of Rh7 did not further impair re-synchronization in flies lacking *norpA* and *cry* function, indicating that the phenotypes described earlier are specific for Rh1, Rh5, Rh6, and Gq [27]. To better visualize re-synchronization of the activity peaks, we also plotted the average daily activity before the shift and during the final 3 days before release into DD next to the actograms. While, in *norpA^{P41} cry^{b}* flies, the main evening activity peak before and after the shift occurs almost at the same time with relation to the LD cycle, the activity peak in the various rhodopsin and Gq mutants does not shift or only shifts minimally (Figures 2A–2D). We also quantified this behavior by determining the daily shift of the activity peak, again revealing no, or minimal, behavioral adjustment to the delayed LD cycle in the rhodopsin and Gq mutants (Figure 2E; STAR Methods).

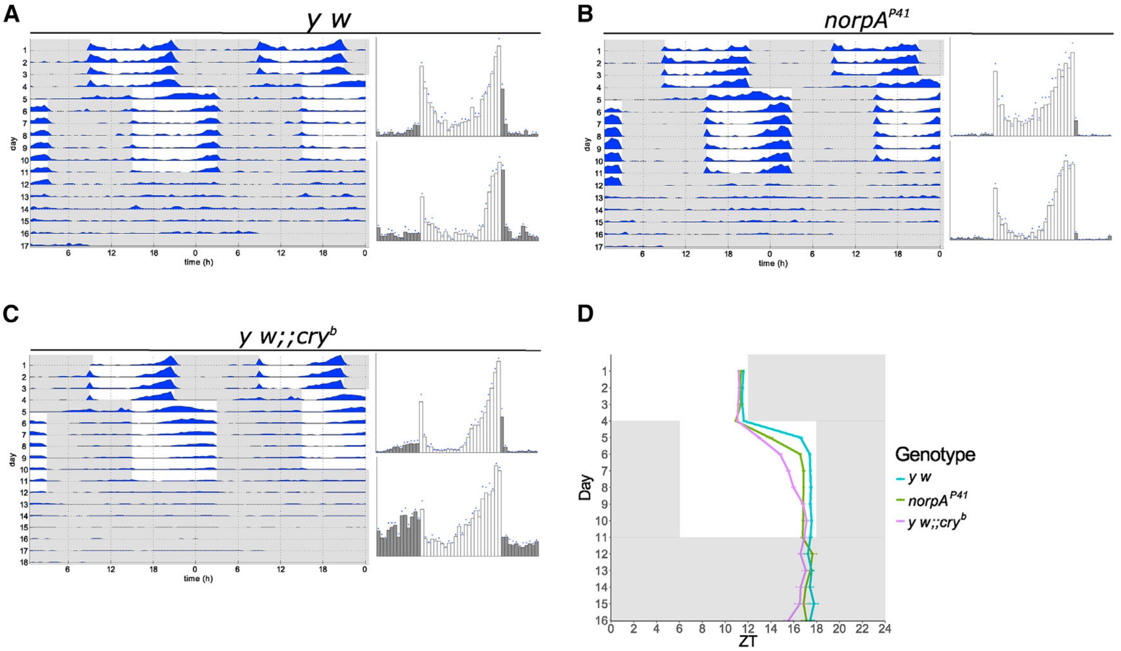

Figure 1. *norpA* and *cry* Mutants Re-entrain More Slowly than Wild-Type

(A–C) Representative actograms (left) of 16 flies for each of the genotypes and histograms (right) of the last 3 days before the shift (top) and after the shift (bottom). (A) *y w*. (B) *norpA*^{P41}. (C) *y w;; cry*^{b}.

(D) Quantification of the position of the evening peaks for the three genotypes for each day of the experiment. While wild-type flies move their evening peak by 5 hr on the first day and 0.8 hr on the second day of the phase shift, *norpA*^{P41} mutants need almost 2 days to move their evening peak by 5.6 hr (3 hr on the first day and 2.6 hr on the second). *cry* mutants shift 5.6 hr in 5 days (1.9 hr on the first day, 1.8 hr on the second, 0.7 hr on the third, 0.4 hr on the fourth, and 0.8 hr on the fifth). *y w*, n = 58; *norpA*^{P41}, n = 31; *y w;; cry*^{b}, n = 56.

See also Figure S1.

norpA- and *cry*-Independent Light Signaling Targets the s-LNv Pacemaker Neurons

The ability of *norpA*^{P41} *cry*^{b} double mutants to resynchronize activity rhythms to LD cycles is presumably mediated by molecular oscillations in at least some of the clock neurons that are known to control these rhythms. Indeed, a previous study showed that PER oscillations in the s-LNvs of *norpA*^{P41} *cry*^{b} flies are synchronized during LD cycles [20]. Therefore, we determined whether PER oscillations could be synchronized by LD cycles (at constant temperature) in clock neurons of *norpA*^{P41} *cry*^{b} flies and in flies that additionally lacked rhodopsins or Gq. In agreement with the previous study [20], *norpA*^{P41} *cry*^{b} double mutants maintain robust PER oscillations during the LD cycle in the s-LNvs. Indeed, we show here that, in contrast to the l-LNvs, the four PDF-expressing s-LNvs and the fifth PDF-negative s-LNv maintain light-synchronized oscillations in *norpA*^{P41} *cry*^{b} (Figures 3A–3D). In contrast, flies lacking additionally Rh5 and Rh6, as well as those lacking Gq, do not show rhythmic accumulation of PER, presumably due to the lack of light-mediated degradation during the day (see high PER levels at zeitgeber time [ZT] 10 in Figure 3). We also analyzed PER levels in s-LNvs at normal peak (ZT22) and trough (ZT10) time points in flies lacking Rh1, in addition to PLC-β and CRY, and again saw maintained high PER levels at ZT10, suggesting a block of light-dependent degradation (Figure 3). We also determined PER levels in the other clock neuronal groups but did not observe any consistent differences between *norpA*^{P41} *cry*^{b} flies and those lacking rhodopsins or Gq in addition (Figure S2). These results strongly suggest that the main target for the *norpA*- and *cry*-independent light input are the PDF^{+} and PDF^{-} s-LNvs and that light-synchronized oscillations in these neurons are responsible for the behavioral synchronization of *norpA*^{P41} *cry*^{b} flies.

Rh5 and Rh6 Dominate the Residual ERG Responses in *norpA* Mutant Flies

Our results indicate a *norpA*- and *cry*-independent role for Rh1, Rh5, Rh6, and Gq in behavioral and molecular light resetting of the *Drosophila* clock. All of these genes and proteins are expressed in the retinal photoreceptors; Rh1 is expressed in the outer photoreceptor cells R1–R6; Rh5 and Rh6 are expressed in the inner R8 cells; and Gq is expressed in all photoreceptors. In addition Rh6 is expressed in the H-B eyelet, e.g [35], but although the Rh5 promoter is also active in this structure, Rh5 protein could not be detected in the eyelet [25, 28]. Therefore, we speculated that the most relevant tissue for the circadian clock function of Rh1, Rh5, Rh6 and Gq was the retina. In an attempt to demonstrate the involvement of retinal rhodopsins in *norpA*-independent phototransduction, we performed ERG recordings with flies lacking *norpA* as well as several rhodopsins and Gq. All genotypes carrying *norpA*^{P41} were also mutant for *cry* (i.e., they were the same lines we used for the behavioral analysis), though it has recently been reported that the lack of CRY does not influence the ERG [36].

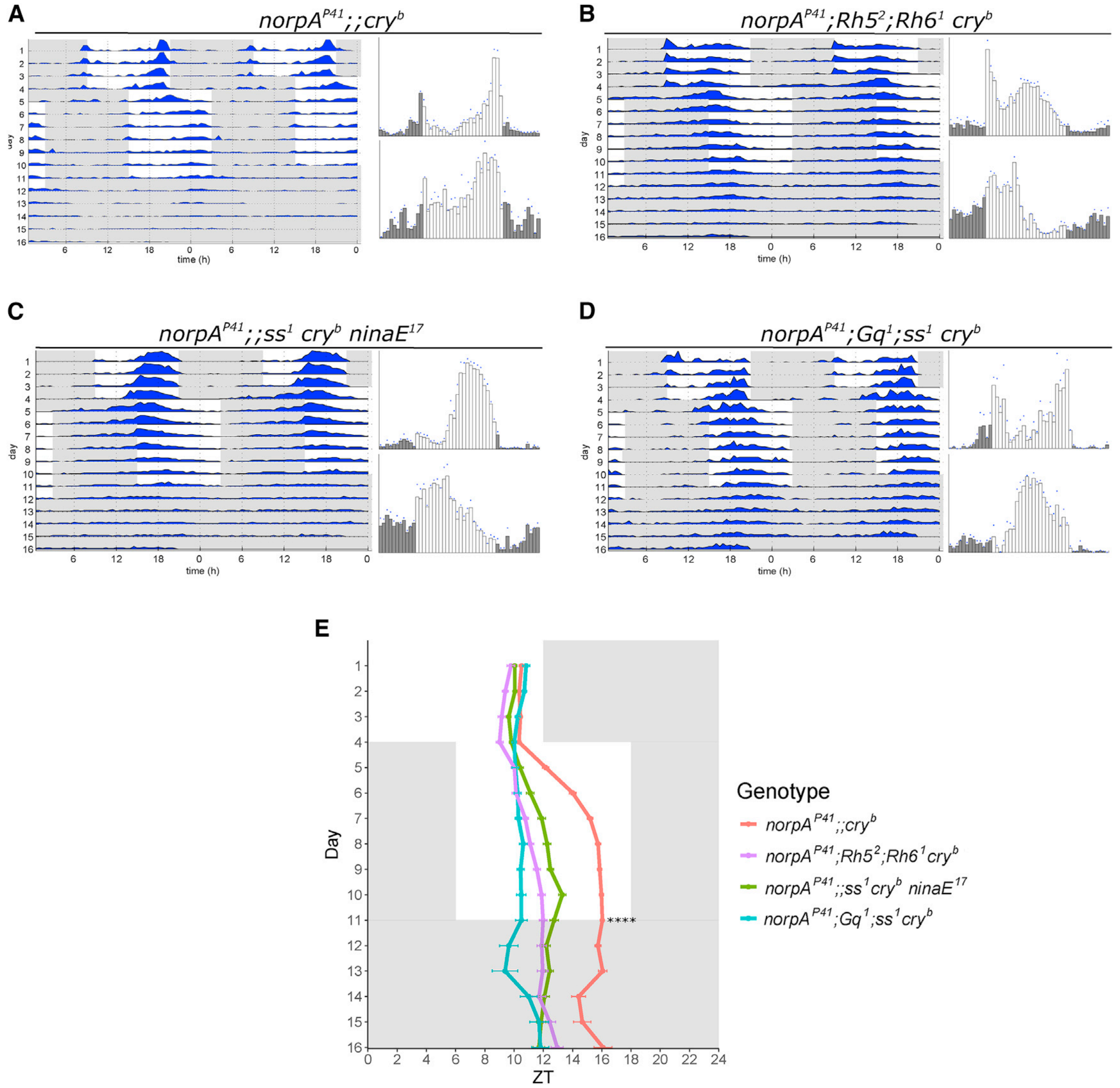

Figure 2. Rh1, Rh5, Rh6, and Gq Contribute to *norpA*- and *cry*-Independent Behavioral Light Resetting

(A–D) Representative actograms (left) of 16 flies for each of the genotypes, and histograms (right) of the last 3 days before the shift (top) and after the shift (bottom). Flies were entrained to a 2-hr ramping 12-hr:12-hr LD cycle in combination with temperature cycles of 25:16°C. After 4 days, the temperature was kept constant at 25°C, and the LD regime was shifted 6 hr. The flies were kept in these new conditions for 7 days, and afterward they were in constant darkness for 5 days. (A) $norpA^{P41}$;; cry^b. (B) $norpA^{P41}$; $Rh5^2$; $Rh6^1$ cry^b. (C) $norpA^{P41}$;; ss^1 cry^b $ninaE^{17}$. (D) $norpA^{P41}$; Gq^1; ss^1 cry^b.

(E) Comparison of the position of the evening peak for each of the genotypes for each of the days of the experiment. While $norpA^{P41}$;; cry^b flies shift their evening peak by 5.7 ± 0.2 hr during the 7 days of the new regime, none of the other mutants are able to adapt to the new conditions ($norpA^{P41}$; $Rh5^2$; $Rh6^1$ cry^b, 3.0 ± 0.3 hr; $norpA^{P41}$; Gq^1; cry^b, 0.5 ± 0.4 hr; and $norpA^{P41}$;; $ninaE^{17}$ cry^b, 2.9 ± 0.3 hr). $norpA^{P41}$;; cry^b, n = 40; $norpA^{P41}$; $Rh5^2$; $Rh6^1$ cry^b, n = 55; $norpA^{P41}$; Gq^1; ss^1 cry^b, n = 41; and $norpA^{P41}$;; ss^1 cry^b $ninaE^{17}$, n = 57.

Error bars represent SEM. ****p < 0.0000001.

See also Figures S1A, S1B, and S3.

$norpA^{P41}$ is a true loss-of-function allele [28] and was reported to lack all ERG responses to light [37] (Figure 4A). Upon closer inspection, however, small (<1 mV) ERG responses to bright illumination are detectable in $norpA^{P41}$ mutant flies (Figures 4B and 4C). As an extracellular potential, photoreceptor depolarization in the ERG is manifested as a maintained hyperpolarizing (negative) response, while positive-going "on" and negative-going "off" transients reflect transient activation of postsynaptic interneurons. In $norpA^{P41}$, a small maintained hyperpolarization of up to ~1 mV was detected in response to a 2-s bright step of light, which then decayed very slowly (Figures 4B, 4C, and S1C). Surprisingly, this response was largely unaffected (if anything, slightly enhanced) in $norpA^{P41}$ $ninaE^{17}$ double mutants lacking visual pigment in the major R1–R6 class (Figures 4B and 4C). By contrast, in $norpA^{P41}$ $Rh5^2$ $Rh6^1$ treble mutants lacking visual pigment in all R8 cells, the negative-going ERG was largely abolished, leaving a small, predominantly positive-going response (Figures 4B and 4C). This suggests that the R8 cells contribute

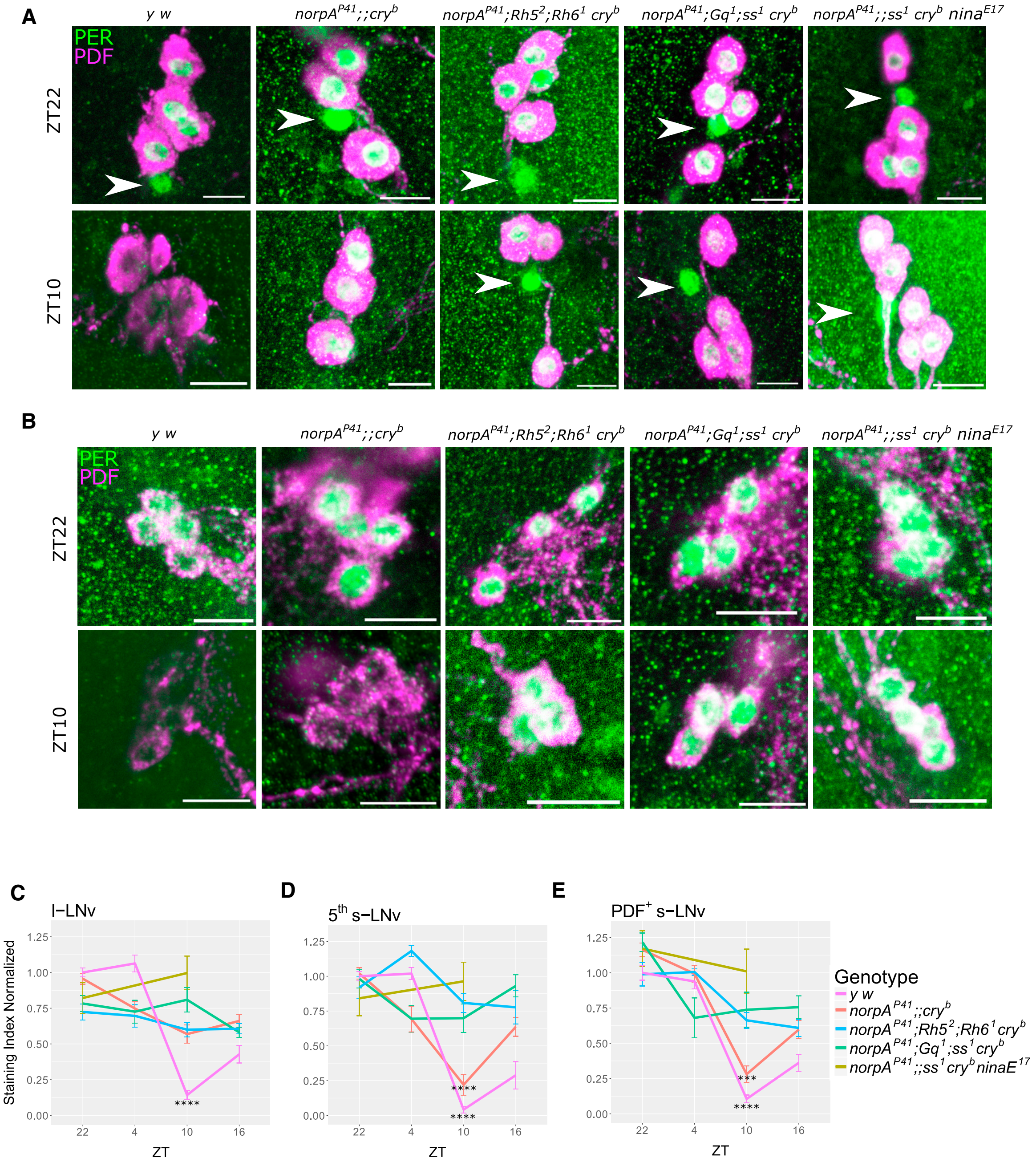

Figure 3. Rh1, Rh5, Rh6, and Gq Are Required for Synchronized PER Oscillations in the s-LN Clock Neurons

(A and B) Representative images of the staining at ZT22 and ZT10 of the l-LNvs and the fifth s-LNv (A; fifth s-LNv marked with arrowheads) and the s-LNvs (B), with PER antibody indicated in green and PDF antibody indicated in magenta to identify the fifth PDF-negative s-LNv. Scale bars, 10 μm.

(C–E) Quantification of PER expression in the l-LNv (C), fifth s-LNv (D), and PDF$^+$ s-LNv (E). Note that PER oscillations in the PDF$^+$ and PDF$^-$ s-LNvs are synchronized to the LD cycle in *y w* and *norpA^{P41};; cry^b* flies, but not in any of the other genotypes, presumably because of PER is not degraded during the day (compare PER values at ZT10 between the genotypes: PER levels in *y w* and *norpA^{P41};; cry^b* s-LNvs are significantly lower compared to any of the mutants; ****p < 0.0000001 and ***p < 0.0001, respectively). For ZT22 and ZT10 time points, between 12 (*norpA^{P41};; ninaE^{17} ss^1 cry^b*) and 51 (*y w*) brain hemispheres were imaged in 2–5 independent experiments. For ZT4 and ZT16 time points, between 6 (*norpA^{P41}; Gq^1; ss^1 cry^b*) and 19 (*y w*) hemispheres were analyzed in 3 independent experiments (see Table S1 for exact numbers).

Error bars indicate SEM.

See also Figure S2 and Table S1.

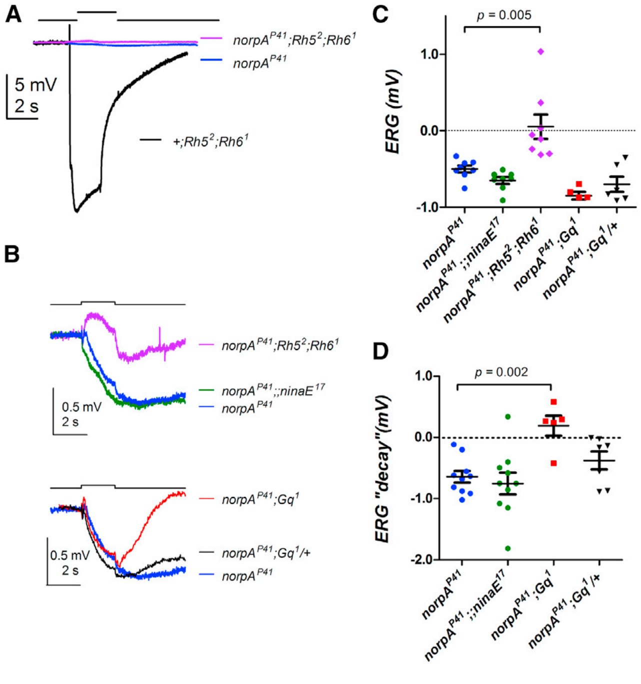

Figure 4. Rh5 and Rh6 Dominate the *norpA*-Independent ERG Responses

(A) ERG in response to a 2-s bright white light flash (bar, equivalent to ~10^7 effective photons per photoreceptor) in *norpA^{P41}* (blue), *Rh5^2; Rh6^1* (black), and *norpA^{P41}; Rh5^2; Rh6^1* (purple) mutants.

(B) ERG responses in response to a 2-s bright white flash. Top traces recorded from *norpA^{P41}* flies (blue), *norpA^{P41} ; Rh5^2; Rh6^1* flies lacking both R8 opsins (purple), and *norpA^{P41};; ninaE^{17}* mutant flies lacking the R1–R6 opsin (green). Lower traces recorded from *norpA^{P41}* flies (blue; same as upper panel), *norpA^{P41}*; *Gq^1* flies (red), and *norpA^{P41}*; *Gq^1/CyO* flies (black; sibling controls from same vials). Each trace is a "grand" average from responses from 5 to 10 flies, each of which was itself averaged from responses to at least 4 flashes repeated at 5-min intervals.

(C) Summary of response amplitudes averaged over the last 500 ms of the 2-s flash. Responses in *norpA^{P41} ; Rh5^2; Rh6^1* were significantly (p = 0.005) more positive than *norpA^{P41}* controls. Other genotypes were not significantly different (one-way ANOVA with Dunnett's multiple comparison).

(D) Summary of response amplitudes averaged between 2 and 4 s after termination of the stimulus. Responses in *norpA^{P41}*; *Gq^1* recovered significantly more quickly (p = 0.002). All genotypes were also homozygous for the *cry^b* allele, but CRY does not influence the ERG response [36].

See also Figure S1C.

most of the receptor component of the residual ERG in *norpA^{P41}* flies. ERG responses in *norpA^{P41} Gq^1* were similar in amplitude compared to *norpA^{P41}* alone, but the duration of the response was significantly shortened (Figures 4B and 4D). This shows that Gq influences the duration of the *norpA*-independent residual ERG response, in line with observations from whole-cell patch-clamp recordings from R1–R6 photoreceptors [38]. While the underlying physiological mechanisms for these effects are not known, the results, nevertheless, indicate an involvement of Rh5, Rh6, and Gq in the *norpA*-independent ERG response.

PLC21C-Encoded PLC-β Participates in Circadian Light Resetting and ERG Responses

A candidate for the alternative Gq target is PLC21C, the second PLC-β encoded in the *Drosophila* genome. PLC21C is enriched in heads and the CNS [39], and we show here that *Plc21C* mRNA is also expressed in the retina (Figure 5A). To test whether *Plc21C* participates in *norpA*-independent circadian photoreception, we combined the *Plc21C^{P319}* allele with *norpA^{P41}* and *cry^b*. *Plc21C^{P319}* is a strong hypomorphic allele, which drastically reduces the amplitude of electroantennogram recordings in response to odor stimulation [39, 40]. Surprisingly, the treble-mutant flies did not synchronize to shifted LD cycles, similar to introducing *Gq^1*, *ninaE^{17}*, or *Rh5^2 Rh6^1* into the same background (Figures 5B and 5D; compare to Figure 2). To confirm that *Plc21C* and *Gq* operate in the same pathway, we also tested the light-resetting ability of transheterozygous *norpA^{P41} Gq^1/Plc21C^{P314} cry^b* flies. Although these flies carry one wild-type copy of the *Gq* and *Plc21C* genes, they are not able to resynchronize to the shifted LD cycle, demonstrating that both genes genetically interact and strongly indicating that Gq activates PLC21C (Figures 5C and 5D). The same strategy (with identical alleles) has been used previously to demonstrate that Gq activates Plc21C in the olfactory system [41]. Next, we performed ERG recordings from *norpA^{P41} Plc21C^{P319} cry^b* flies and found that, similar to *norpA^{P41} Rh5^2 Rh6^1 cry^b* flies, they only showed a small positive-going response to brief light pulses (Figures 5E and 5F). These data suggest that *Plc21C* contributes to circadian light resetting and *norpA*-independent ERG response, presumably in response to Rh5/Rh6-mediated activation of Gq.

DISCUSSION

The daily environmental changes of light and darkness arguably represent the most reliable circadian clock resetting cue for all organisms exposed to these natural LD cycles. Not surprisingly, various photoreceptors and photopigments contribute to light synchronization in plants and animals, with multiple light-input pathways existing within one species, e.g [20, 41, 42]. In *Drosophila,* next to the well-established role of the blue light photoreceptor CRY, expressed within subsets of the circadian clock neurons, the visual system also contributes to daily light resetting, e.g [4, 20]. Contribution of the visual system involves canonical rhodopsin signaling, which depends on *norpA*-encoded PLC-β, and which is important for synchronization to low-light LD cycles [4, 22]. On the other hand, Rh5 and Rh6 have been shown to be part of a *norpA*-independent pathway, contributing to the synchronization of medium-intensity-light LD cycles (~400 lux) and suggesting the existence of non-canonical rhodopsin phototransduction in *Drosophila* [28]. Here, we demonstrate that *Rh1*, *Rh5*, *Rh6*, *Gq*, and *Plc21C* are required for light synchronization of molecular and behavioral circadian rhythms in the absence of CRY and canonical visual phototransduction.

Molecular Synchronization by Light in the Absence of CRY and Canonical Phototransduction

Ultimately, all resetting cues that have the potency to stably synchronize clock-controlled behavioral rhythms must affect the molecular oscillations in at least a subset of the clock neurons. The main target of light signals is the clock protein TIM, which is degraded after light-dependent interaction with the photoreceptor CRY and the F-box protein JET [5, 8–10]. However, CRY is only expressed in about 50% of the ~150 clock

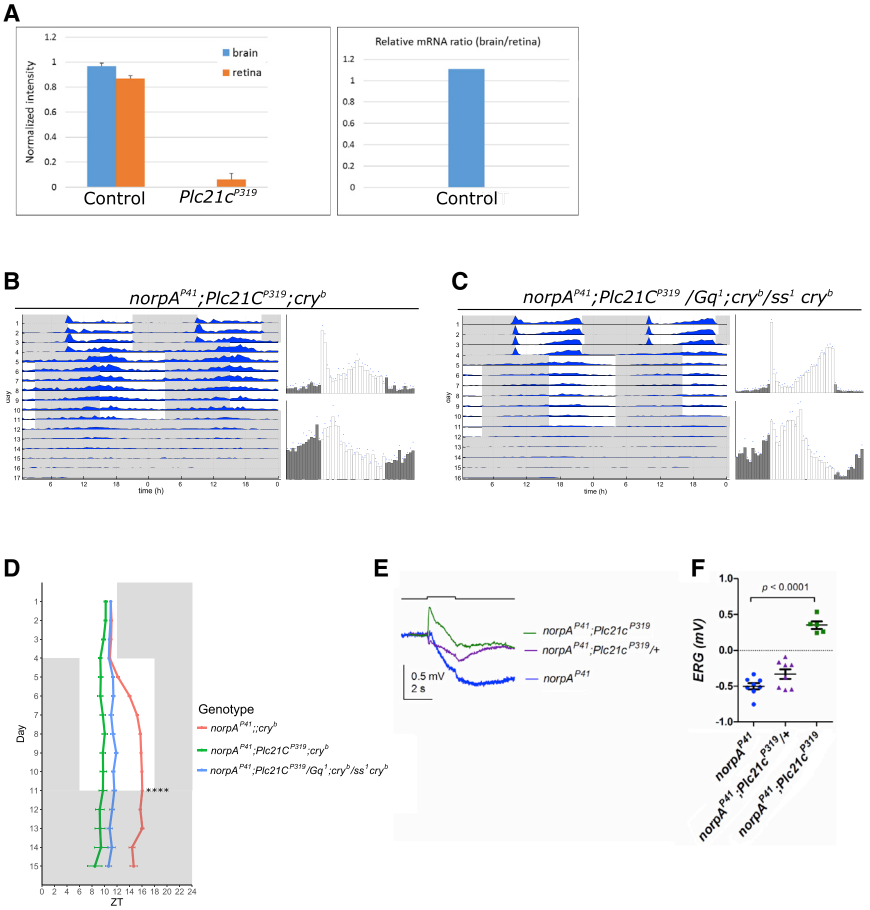

Figure 5. PLC21C Participates in Circadian Light Resetting and ERG Responses

(A) Determination of *Plc21C* expression levels in brains and retinas of control and mutant flies by semi-qRT-PCR. Data were normalized against the ribosomal gene *rp49*.

(B and C) Representative actograms (left) of 16 flies for each of the genotypes, and histograms (right) of the last 3 days before the shift (top) and after the shift (bottom). (B) *norpA*^{P41}; *Plc21C*^{P319}; *cry*^{b}. (C) *norpA*^{P41}; *Plc21C*^{P319}/*Gq*^{1}; *cry*^{b}/ *ss*^{1} *cry*^{b}.

(D) Phase determination of evening activity peaks for *norpA*^{P41};; *cry*^{b} ($n = 40$, same as in Figure 2), *norpA*^{P41}; *Plc21C*^{P319}; *cry*^{b} ($n = 36$), and *norpA*^{P41}; *Plc21C*^{P319}/*Gq*^{1}; *cry*^{b}/ *ss*^{1} *cry*^{b} ($n = 28$) flies. In the 7 days of the new light regime, neither *norpA*^{P41}; *Plc21C*^{P319}; *cry*^{b} flies nor *norpA*^{P41}; *Plc21C*^{P319}/ *Gq*^{1}; *cry*^{b}/ *ss*^{1} *cry*^{b} flies are able to shift their main activity peak.

(E) ERG in response to a 2-s bright white light flash in *norpA*^{P41} (blue), *norpA*^{P41}; *Plc21C*^{P319}/+; *cry*^{b} (purple), and *norpA*^{P41}; *Plc21C*^{P319}; *cry*^{b} (green) flies. Each trace is a "grand" average from responses from 5–10 flies, each of which was itself averaged from responses to at least 4 flashes repeated at 5-min intervals.

(F) Summary of response amplitudes averaged over the last 500 ms of the 2-s flash. *norpA*^{P41}; *Plc21C*^{P319}; *cry*^{b} no longer showed any hyperpolarizing component at this state ($p < 0.0001$, one-way ANOVA with Dunnett's multiple comparison). *norpA*^{P41}; *Plc21C*^{P319}/+ heterozygotes also showed a reduced hyperpolarizing component, though this was only marginally significant on this sample ($p = 0.06$).

See also Figure S1.

neurons in the fly brain [43, 44], and light-dependent synchronization of clock protein cycling still occurs in some of clock neurons in *cry* mutants, including the s-LNv, LNd, and DN1 neurons that normally express *cry* [4, 20, 23, 30–32, 45]. Furthermore, TIM is CRY-independently degraded in s-LNv and LNd clock neurons after artificial excitation of the PDF^{+} LNv [46]. This degradation requires PDF signaling from s-LNv to LNd and the E3 ubiquitin ligase CUL-3, which is also required for TIM degradation in constant darkness [46, 47]. Taken together, these findings show that both light-dependent and light-independent TIM degradation mechanisms exist, which do not depend on CRY and, presumably, are responsible for the synchronized TIM and PER oscillations in *norpA cry* double mutants. Here, we reveal that light-synchronized PER oscillations in the four PDF^{+} s-LNvs and the PDF^{-} fifth s-LNv are abolished by blocking *norpA*- and *cry*-independent photoreception. In other words, we show that Rh1, Rh5, Rh6, and Gq are part of the CRY-independent molecular resetting mechanism targeting the s-LNv pacemaker neurons. Interestingly, CRY-independent light-synchronized oscillations in the fifth s-LNv, but not in the PDF^{+} s-LNvs, depend on PDF signaling [30, 32, 45]. This means that the rhodopsin- and Gq-dependent (but *norpA*-independent) mechanism signals light information most likely directly to the PDF^{+} s-LNv, resulting in CRY-independent molecular clock resetting in all s-LNvs.

Role of PLC21C in Rhodopsin-Mediated Phototransduction

We show here that the *norpA*-independent pathway depends on Gq, which is also central to the canonical phototransduction pathway. This means that downstream of Gq activation by Rh5, Rh6, and Rh1, alternative effector molecules signal light information to the circadian clock neurons. Our data suggest that the alternative Gq target is PLC21C, the second PLC-β encoded in the fly genome [39]. This is a surprising finding, because PLC21C has not previously been implicated in photoreceptor function, although it has been shown to function in olfactory signal transduction in the antennae downstream of Gq [40]. In addition, *Plc21c* is expressed in the CNS and, presumably, within subsets of the clock neurons (the PDF expressing LNv), where this enzyme has recently been implicated in light resetting of the circadian clock by brief light pulses [26]. Ni et al. (2017) suggest that PLC21C is activated by Rh7, which, they show, is expressed in the LNv contributing to clock resetting after exposure to brief light pulses. Nevertheless, Rh7 mutants do not show any re-synchronization defects when exposed to shifted white-light LD cycles, as we applied here [27]. Rh7 mutants also did not increase the synchronization deficits of single *cry* or *norpA cry* double mutants [27], showing that Rh7 does not contribute to *norpA*-independent light resetting. Moreover, PLC21C has been shown to act downstream of Go

in LNvs to set the (light-independent) free-running period length to 24 hr in response to GABAergic input [48]. Considering the LNv-specific and light-independent functions associated with PLC21C, combined with its known role in olfactory sensory neurons, the new role for this enzyme in the *norpA*-independent light-input pathway was unexpected and warrants further investigation.

Which Photoreceptor Cells Utilize the Novel Transduction Pathway?

Based on our behavior and molecular light-resetting results, it is likely that the Rh1-expressing photoreceptor cells R1–R6, as well as the Rh5- and Rh6-expressing R8 photoreceptors, contribute to *norpA*-independent clock resetting. It is also possible that the R7 cells expressing the UV-sensitive opsins Rh3 and Rh4, as well as blue-light-sensitive Rh2-expressing ocelli, participate in *norpA*-independent light synchronization, but to test their contribution, experiments need to be performed with the specific light spectra in contrast to the white LED light regime used in the present study.

The neuronal connection between the retinal photoreceptors and the circadian clock neurons is currently unknown, but presumably is indirect; e.g [12]. In addition to the R8 cells, 4 photoreceptive cells constituting the H-B eyelet express Rh6 and have been implicated in light signaling to the circadian clock [20, 23, 25, 28]. In fact, a recent study revealed direct synaptic contacts between the H-B eyelet axons and the dendritic fields of the LNv neurons [35]. Moreover, artificial excitation of all Rh6-expressing cells resulted in Ca^{2+} and cyclic AMP (cAMP) increases in the PDF^{+} s-LNv, but not the l-LNv, consistent with our results showing that Rh6 contributes to molecular clock resetting in the s-LNv [35] (Figure 3). Therefore, we conclude that, most likely, both retinal and H-B eyelet photoreceptors utilize *norpA*-independent light signaling, targeting the s-LNv.

norpA-Independent Features of the ERG Responses

Removal of Rh5 and Rh6 function strongly attenuated the residual hyperpolarizing component of the ERG responses of *norpA* mutants to bright white-light flashes, suggesting that the R8 cells substantially contribute to the remaining light sensitivity in the absence of *norpA*. It also suggests that this remaining sensitivity contributes to both behavioral and molecular light resetting of the circadian clock (Figures 2 and 3). Removal of Gq also affected the *norpA* ERG: although the kinetics and degree of the initial negative response, reflecting depolarization of the (R8) photoreceptor cells, were similar in *norpA* single and *norpA Gq* double mutants, responses lasted considerably longer in the single mutants (Figures 4B and 4D). Although we would have expected a stronger effect on the initial response, our results show that a Gq-dependent component contributes to the residual light responses of *norpA* mutants, and it is possible that this Gq function correlates with the role of Gq in behavioral and molecular synchronization (Figuress 2 and 3). The weaker effects (compared to Rh5 and Rh6 removal) could be explained by the fact that the Gq^{1} allele is a severe hypomorph [49], whereas $Rh5^{2}$ and $Rh6^{1}$ alleles are true loss-of-function mutants. Also, we cannot rule out the possibility that part of the residual *norpA* ERG sensitivity is independent of Gq, although the severe phenotype observed in *Plc21C* mutants suggests that it solely depends on Gq activation by Rh5 and Rh6.

Requirement of Rh1, Rh5, and Rh6 for Molecular and Behavioral Synchronization

Surprisingly, removal of Rh1 did not noticeably alter the residual *norpA* ERG response (Figure 4B), suggesting that the fly's major rhodopsin signals almost exclusively via *norpA*-encoded PLC-β. But why did we observe a *norpA*-independent function for *Rh1* in behavioral and molecular synchronization (Figures 2 and 3)? The $ninaE^{17}$ cry^{b} and Gq^{1} cry^{b} flies also carry the *spineless*^{1} (ss^{1}) allele, and loss-of-function *ss* alleles have been shown to drastically reduce *Rh4* and *Rh6* expression in R7 and R8 cells, respectively [50]. In principle, this raises the possibility that the lack of synchronization observed in both genotypes could be caused by a reduction in Rh6 rather than the loss of Rh1 or reduction in Gq. However, ss^{1} is a weak allele, and although Rh6 expression has not been investigated, it shows only a mild reduction of Rh4 expression in R7 cells (data not shown); also, it does not show the antennal transformations and tarsal deletions associated with strong *ss* alleles [51]. In addition, we found no difference in the slow-synchronization phenotype between $norpA^{P41}$ cry^{b} mutants with and without ss^{1} (Figure S3), unlike $norpA^{P41}$ $Rh6^{1}$ cry^{b} mutants, which show a decrease in synchronization speed compared to $norpA^{P41}$ cry^{b} [20, 29]. Taken together, we are therefore confident that the behavioral and molecular synchronization defects observed in $norpA^{P41}$ $ninaE^{17}$ ss^{1} cry^{b} and $norpA^{P41}$ Gq^{1} ss^{1} cry^{b} flies are, indeed, caused by the lack or reduction of Rh1 and Gq, respectively.

Although we see a correlation between the circadian assays and the ERG recordings with regard to Rh5, Rh6, Gq, and PLC21C function, it is questionable to what extent results obtained from the two assays are related. For example, ERGs are responses measured to a 2-s flash of bright light, whereas the circadian assays apply 12-hr:12-hr ramped LD cycles, with light intensities ranging from 0 to 180 lux. There could, however, be a physiologically more interesting explanation for the discrepancy that we observe between the experiments involving removal of Rh1. Namely, the lack of molecular and behavioral synchronization suggests that that integration of signals from both R1–R6 and R8 is required for circadian clock entrainment to occur. This might be implemented either by a common downstream interneuron or, for example, by R1–R6 signals being fed into R8, which would then, in effect, also be acting as an interneuron (although these signals would presumably have to be ''silent'' at the level of the ERG to explain the lack of effect in the $norpA^{P41}$ $ninaE^{17}$ double mutant). Such a situation would have interesting parallels with circadian entrainment in mammals, which is mediated both by melanopsin-expressing, intrinsically light-sensitive retinal ganglion cells (RGCs) and by the rods and cones [42]. Interestingly, the RGCs act both as photoreceptor cells and as interneurons for propagating the rod and cone signals.

Conclusions

We have shown that non-canonical phototransduction involving Rh1, Rh5, Rh6, Gq, and PLC21C can contribute to circadian

clock resetting in *Drosophila* in the absence of NORPA. This novel pathway targets a specific subgroup of clock neurons, the s-LNvs, and synchronizes clock protein oscillations in these cells independent of the cell-autonomous circadian photoreceptor CRY. Remarkably, except for Rh1, the same factors also contribute to residual, *norpA*-independent, visual system light sensitivity to brief flashes of light, highlighting the versatility and biological significance of this novel pathway.

STAR★METHODS

Detailed methods are provided in the online version of this paper and include the following:

- KEY RESOURCES TABLE
- CONTACT FOR REAGENT AND RESOURCE SHARING
- EXPERIMENTAL MODEL AND SUBJECT DETAILS
  - Flies
- METHOD DETAILS
  - Behavior analysis
  - Electroretinogram recordings
  - Immunohistochemistry
- QUANTIFICATION AND STATISTICAL ANALYSIS
- DATA AND SOFTWARE AVAILABILITY

SUPPLEMENTAL INFORMATION

Supplemental Information includes three figures and one table and can be found with this article online at https://doi.org/10.1016/j.cub.2018.04.016.

ACKNOWLEDGMENTS

We thank Charlotte Helfrich-Förster, Gaiti Hasan, Claude Desplan, and Francois Rouyer for fly stocks and Luis Garcia for developing the programmable LED system. This work was supported by Biotechnology and Biological Sciences Research Council grant BB/J018589/2 and by the European Union (FP7 Integrated Training Network INSecTIME: PITN-GA-2012-316790 to R.S. and a Marie Curie WHRI-Academy COFUND fellowship: PCOFUND-GA-2013-608765 to M.O.) and a Biotechnology and Biological Sciences Research Council grant BB/M007006/1 to R.C.H.

AUTHOR CONTRIBUTIONS

M.O. performed, analyzed, and interpreted all behavioral and immunohistological experiments. R.C.H. performed, analyzed, and interpreted all ERG experiments. R.S. designed and interpreted experiments and wrote the paper (with help from M.O. and R.C.H).

DECLARATION OF INTERESTS

The authors declare no competing interests.

Received: February 14, 2018
Revised: March 23, 2018
Accepted: April 4, 2018
Published: May 17, 2018

REFERENCES

1. Chaix, A., Zarrinpar, A., and Panda, S. (2016). The circadian coordination of cell biology. J. Cell Biol. *215*, 15–25.
2. Hardin, P.E. (2011). Molecular genetic analysis of circadian timekeeping in Drosophila. Adv. Genet. *74*, 141–173.
3. Emery, P., So, W.V., Kaneko, M., Hall, J.C., and Rosbash, M. (1998). CRY, a *Drosophila* clock and light-regulated cryptochrome, is a major contributor to circadian rhythm resetting and photosensitivity. Cell *95*, 669–679.
4. Stanewsky, R., Kaneko, M., Emery, P., Beretta, B., Wager-Smith, K., Kay, S.A., Rosbash, M., and Hall, J.C. (1998). The *cry*^{b} mutation identifies cryptochrome as a circadian photoreceptor in *Drosophila*. Cell *95*, 681–692.
5. Busza, A., Emery-Le, M., Rosbash, M., and Emery, P. (2004). Roles of the two *Drosophila* CRYPTOCHROME structural domains in circadian photoreception. Science *304*, 1503–1506.
6. Czarna, A., Berndt, A., Singh, H.R., Grudziecki, A., Ladurner, A.G., Timinszky, G., Kramer, A., and Wolf, E. (2013). Structures of *Drosophila* cryptochrome and mouse cryptochrome1 provide insight into circadian function. Cell *153*, 1394–1405.
7. Dissel, S., Codd, V., Fedic, R., Garner, K.J., Costa, R., Kyriacou, C.P., and Rosato, E. (2004). A constitutively active cryptochrome in *Drosophila melanogaster*. Nat. Neurosci. *7*, 834–840.
8. Koh, K., Zheng, X., and Sehgal, A. (2006). JETLAG resets the *Drosophila* circadian clock by promoting light-induced degradation of TIMELESS. Science *312*, 1809–1812.
9. Peschel, N., Veleri, S., and Stanewsky, R. (2006). Veela defines a molecular link between Cryptochrome and Timeless in the light-input pathway to Drosophila's circadian clock. Proc. Natl. Acad. Sci. USA *103*, 17313–17318.
10. Peschel, N., Chen, K.F., Szabo, G., and Stanewsky, R. (2009). Light-dependent interactions between the *Drosophila* circadian clock factors *cryptochrome, jetlag*, and *timeless*. Curr. Biol. *19*, 241–247.
11. Peschel, N., and Helfrich-Förster, C. (2011). Setting the clock–by nature: circadian rhythm in the fruitfly *Drosophila melanogaster*. FEBS Lett. *585*, 1435–1442.
12. Muraro, N.I., and Ceriani, M.F. (2015). Acetylcholine from Visual Circuits Modulates the Activity of Arousal Neurons in *Drosophila*. J. Neurosci. *35*, 16315–16327.
13. Parisky, K.M., Agosto, J., Pulver, S.R., Shang, Y., Kuklin, E., Hodge, J.J., Kang, K., Liu, X., Garrity, P.A., Rosbash, M., and Griffith, L.C. (2008). PDF cells are a GABA-responsive wake-promoting component of the *Drosophila* sleep circuit. Neuron *60*, 672–682.
14. Shang, Y., Griffith, L.C., and Rosbash, M. (2008). Light-arousal and circadian photoreception circuits intersect at the large PDF cells of the *Drosophila* brain. Proc. Natl. Acad. Sci. USA *105*, 19587–19594.
15. Sheeba, V., Fogle, K.J., Kaneko, M., Rashid, S., Chou, Y.-T., Sharma, V.K., and Holmes, T.C. (2008). Large ventral lateral neurons modulate arousal and sleep in Drosophila. Curr. Biol. *18*, 1537–1545.
16. Renn, S.C., Park, J.H., Rosbash, M., Hall, J.C., and Taghert, P.H. (1999). A *pdf* neuropeptide gene mutation and ablation of PDF neurons each cause severe abnormalities of behavioral circadian rhythms in *Drosophila*. Cell *99*, 791–802.
17. Grima, B., Chélot, E., Xia, R., and Rouyer, F. (2004). Morning and evening peaks of activity rely on different clock neurons of the *Drosophila* brain. Nature *431*, 869–873.
18. Stoleru, D., Peng, Y., Agosto, J., and Rosbash, M. (2004). Coupled oscillators control morning and evening locomotor behaviour of *Drosophila*. Nature *431*, 862–868.
19. Beckwith, E.J., and Ceriani, M.F. (2015). Communication between circadian clusters: The key to a plastic network. FEBS Lett. *589*, 3336–3342.
20. Helfrich-Förster, C., Winter, C., Hofbauer, A., Hall, J.C., and Stanewsky, R. (2001). The circadian clock of fruit flies is blind after elimination of all known photoreceptors. Neuron *30*, 249–261.
21. Rieger, D., Stanewsky, R., and Helfrich-Förster, C. (2003). Cryptochrome, compound eyes, Hofbauer-Buchner eyelets, and ocelli play different roles in the entrainment and masking pathway of the locomotor activity rhythm in the fruit fly *Drosophila melanogaster*. J. Biol. Rhythms *18*, 377–391.
22. Saint-Charles, A., Michard-Vanhée, C., Alejevski, F., Chélot, E., Boivin, A., and Rouyer, F. (2016). Four of the six *Drosophila* rhodopsin-expressing

photoreceptors can mediate circadian entrainment in low light. J. Comp. Neurol. *524*, 2828–2844.

23. Veleri, S., Rieger, D., Helfrich-Förster, C., and Stanewsky, R. (2007). Hofbauer-Buchner eyelet affects circadian photosensitivity and coordinates TIM and PER expression in *Drosophila* clock neurons. J. Biol. Rhythms *22*, 29–42.
24. Salcedo, E., Huber, A., Henrich, S., Chadwell, L.V., Chou, W.-H., Paulsen, R., and Britt, S.G. (1999). Blue- and green-absorbing visual pigments of *Drosophila*: ectopic expression and physiological characterization of the R8 photoreceptor cell-specific Rh5 and Rh6 rhodopsins. J. Neurosci. *19*, 10716–10726.
25. Helfrich-Förster, C., Edwards, T., Yasuyama, K., Wisotzki, B., Schneuwly, S., Stanewsky, R., Meinertzhagen, I.A., and Hofbauer, A. (2002). The extraretinal eyelet of *Drosophila*: development, ultrastructure, and putative circadian function. J. Neurosci. *22*, 9255–9266.
26. Ni, J.D., Baik, L.S., Holmes, T.C., and Montell, C. (2017). A rhodopsin in the brain functions in circadian photoentrainment in *Drosophila*. Nature *545*, 340–344.
27. Kistenpfennig, C., Grebler, R., Ogueta, M., Hermann-Luibl, C., Schlichting, M., Stanewsky, R., Senthilan, P.R., and Helfrich-Förster, C. (2017). A New Rhodopsin Influences Light-dependent Daily Activity Patterns of Fruit Flies. J. Biol. Rhythms *32*, 406–422.
28. Szular, J., Sehadova, H., Gentile, C., Szabo, G., Chou, W.-H.H., Britt, S.G., and Stanewsky, R. (2012). Rhodopsin 5- and Rhodopsin 6-mediated clock synchronization in *Drosophila* melanogaster is independent of retinal phospholipase C-β signaling. J. Biol. Rhythms *27*, 25–36.
29. Emery, P., Stanewsky, R., Helfrich-Förster, C., Emery-Le, M., Hall, J.C., and Rosbash, M. (2000). *Drosophila* CRY is a deep brain circadian photoreceptor. Neuron *26*, 493–504.
30. Cusumano, P., Klarsfeld, A., Chélot, E., Picot, M., Richier, B., and Rouyer, F. (2009). PDF-modulated visual inputs and cryptochrome define diurnal behavior in *Drosophila*. Nat. Neurosci. *12*, 1431–1437.
31. Yoshii, T., Hermann-Luibl, C., Kistenpfennig, C., Schmid, B., Tomioka, K., and Helfrich-Förster, C. (2015). Cryptochrome-dependent and -independent circadian entrainment circuits in *Drosophila*. J. Neurosci. *35*, 6131–6141.
32. Zhang, L., Lear, B.C., Seluzicki, A., and Allada, R. (2009). The CRYPTOCHROME photoreceptor gates PDF neuropeptide signaling to set circadian network hierarchy in *Drosophila*. Curr. Biol. *19*, 2050–2055.
33. Shen, W.L., Kwon, Y., Adegbola, A.A., Luo, J., Chess, A., and Montell, C. (2011). Function of rhodopsin in temperature discrimination in *Drosophila*. Science *331*, 1333–1336.
34. Yoshii, T., Vanin, S., Costa, R., and Helfrich-Förster, C. (2009). Synergic entrainment of Drosophila's circadian clock by light and temperature. J. Biol. Rhythms *24*, 452–464.
35. Schlichting, M., Menegazzi, P., Lelito, K.R., Yao, Z., Buhl, E., Dalla Benetta, E., Bahle, A., Denike, J., Hodge, J.J., Helfrich-Förster, C., and Shafer, O.T. (2016). A Neural Network Underlying Circadian Entrainment and Photoperiodic Adjustment of Sleep and Activity in *Drosophila*. J. Neurosci. *36*, 9084–9096.
36. Mazzotta, G., Rossi, A., Leonardi, E., Mason, M., Bertolucci, C., Caccin, L., Spolaore, B., Martin, A.J., Schlichting, M., Grebler, R., et al. (2013). Fly cryptochrome and the visual system. Proc. Natl. Acad. Sci. USA *110*, 6163–6168.
37. Pearn, M.T., Randall, L.L., Shortridge, R.D., Burg, M.G., and Pak, W.L. (1996). Molecular, biochemical, and electrophysiological characterization of *Drosophila* norpA mutants. J. Biol. Chem. *271*, 4937–4945.
38. Hardie, R.C., Martin, F., Chyb, S., and Raghu, P. (2003). Rescue of light responses in the *Drosophila* "null" phospholipase C mutant, $norpA^{P24}$, by the diacylglycerol kinase mutant, *rdgA*, and by metabolic inhibition. J. Biol. Chem. *278*, 18851–18858.
39. Shortridge, R.D., Yoon, J., Lending, C.R., Bloomquist, B.T., Perdew, M.H., and Pak, W.L. (1991). A *Drosophila* phospholipase C gene that is expressed in the central nervous system. J. Biol. Chem. *266*, 12474–12480.
40. Kain, P., Chakraborty, T.S., Sundaram, S., Siddiqi, O., Rodrigues, V., and Hasan, G. (2008). Reduced odor responses from antennal neurons of G(q) alpha, phospholipase Cbeta, and *rdgA* mutants in *Drosophila* support a role for a phospholipid intermediate in insect olfactory transduction. J. Neurosci. *28*, 4745–4755.
41. Millar, A.J. (2004). Input signals to the plant circadian clock. J. Exp. Bot. *55*, 277–283.
42. Lucas, R.J., Lall, G.S., Allen, A.E., and Brown, T.M. (2012). How rod, cone, and melanopsin photoreceptors come together to enlighten the mammalian circadian clock. Prog. Brain Res. *199*, 1–18.
43. Benito, J., Houl, J.H., Roman, G.W., and Hardin, P.E. (2008). The blue-light photoreceptor CRYPTOCHROME is expressed in a subset of circadian oscillator neurons in the *Drosophila* CNS. J. Biol. Rhythms *23*, 296–307.
44. Yoshii, T., Todo, T., Wülbeck, C., Stanewsky, R., and Helfrich-Förster, C. (2008). Cryptochrome is present in the compound eyes and a subset of *Drosophila*'s clock neurons. J. Comp. Neurol. *508*, 952–966.
45. Im, S.H., Li, W., and Taghert, P.H. (2011). PDFR and CRY signaling converge in a subset of clock neurons to modulate the amplitude and phase of circadian behavior in *Drosophila*. PLoS ONE *6*, e18974.
46. Guo, F., Cerullo, I., Chen, X., and Rosbash, M. (2014). PDF neuron firing phase-shifts key circadian activity neurons in *Drosophila*. eLife *3*, e02780.
47. Grima, B., Dognon, A., Lamouroux, A., Chélot, E., and Rouyer, F. (2012). CULLIN-3 controls TIMELESS oscillations in the *Drosophila* circadian clock. PLoS Biol. *10*, e1001367.
48. Dahdal, D., Reeves, D.C., Ruben, M., Akabas, M.H., and Blau, J. (2010). *Drosophila* pacemaker neurons require g protein signaling and GABAergic inputs to generate twenty-four hour behavioral rhythms. Neuron *68*, 964–977.
49. Scott, K., Becker, A., Sun, Y., Hardy, R., and Zuker, C. (1995). Gq α protein function in vivo: genetic dissection of its role in photoreceptor cell physiology. Neuron *15*, 919–927.
50. Wernet, M.F., Mazzoni, E.O., Çelik, A., Duncan, D.M., Duncan, I., and Desplan, C. (2006). Stochastic *spineless* expression creates the retinal mosaic for colour vision. Nature *440*, 174–180.
51. Gramates, L.S., Marygold, S.J., Santos, G.D., Urbano, J.-M., Antonazzo, G., Matthews, B.B., Rey, A.J., Tabone, C.J., Crosby, M.A., Emmert, D.B., et al.; the FlyBase Consortium (2017). FlyBase at 25: looking to the future. Nucleic Acids Res. *45* (D1), D663–D671.
52. Stanewsky, R., Frisch, B., Brandes, C., Hamblen-Coyle, M.J., Rosbash, M., and Hall, J.C. (1997). Temporal and spatial expression patterns of transgenes containing increasing amounts of the *Drosophila* clock gene *period* and a *lacZ* reporter: mapping elements of the PER protein involved in circadian cycling. J. Neurosci. *17*, 676–696.
53. O'Tousa, J.E., Baehr, W., Martin, R.L., Hirsh, J., Pak, W.L., and Applebury, M.L. (1985). The *Drosophila ninaE* gene encodes an opsin. Cell *40*, 839–850.
54. Yamaguchi, S., Wolf, R., Desplan, C., and Heisenberg, M. (2008). Motion vision is independent of color in *Drosophila*. Proc. Natl. Acad. Sci. USA *105*, 4910–4915.
55. Cook, T., Pichaud, F., Sonneville, R., Papatsenko, D., and Desplan, C. (2003). Distinction between color photoreceptor cell fates is controlled by Prospero in *Drosophila*. Dev. Cell *4*, 853–864.
56. Sehadova, H., Glaser, F.T., Gentile, C., Simoni, A., Giesecke, A., Albert, J.T., and Stanewsky, R. (2009). Temperature entrainment of *Drosophila*'s circadian clock involves the gene *nocte* and signaling from peripheral sensory tissues to the brain. Neuron *64*, 251–266.
57. Levine, J.D., Funes, P., Dowse, H.B., and Hall, J.C. (2002). Advanced analysis of a *cryptochrome* mutation's effects on the robustness and phase of molecular cycles in isolated peripheral tissues of *Drosophila*. BMC Neurosci. *3*, 5.

58. Ogueta, M., and Garcia Rodriguez, L. (2017). Open Source LED controller for circadian experiments. bioRxiv. https://doi.org/10.1101/201087.

59. Satoh, A.K., Xia, H., Yan, L., Liu, C.-H., Hardie, R.C., and Ready, D.F. (2010). Arrestin translocation is stoichiometric to rhodopsin isomerization and accelerated by phototransduction in *Drosophila* photoreceptors. Neuron *67*, 997–1008.

60. Harper, R.E.F., Dayan, P., Albert, J.T., and Stanewsky, R. (2016). Sensory Conflict Disrupts Activity of the *Drosophila* Circadian Network. Cell Rep. *17*, 1711–1718.

61. Schindelin, J., Arganda-Carreras, I., Frise, E., Kaynig, V., Longair, M., Pietzsch, T., Preibisch, S., Rueden, C., Saalfeld, S., Schmid, B., et al. (2012). Fiji: an open-source platform for biological-image analysis. Nat. Methods *9*, 676–682.

STAR★METHODS

KEY RESOURCES TABLE

REAGENT or RESOURCE	SOURCE	IDENTIFIER
Antibodies		
Rabbit anti-PERIOD	Stanewsky lab, University of Münster [52],	N/A
monoclonal mouse anti-PDF C7	DSHB	Cat# PDF C7, RRID:AB_760350
goat anti-rabbit AlexaFluor 488 nm	Molecular Probes	Cat# A-11034, RRID:AB_2576217
goat anti mouse AlexaFluor 647 nm	Molecular Probes	Cat# A32728, RRID:AB_2633277
Chemicals, Peptides, and Recombinant Proteins		
RNAlater	Ambion	Cat# AM7020
Vectashield	VectorLabs	Cat# H-1000
Critical Commercial Assays		
RNeasy kit	QIAGEN	Cat# 74104
RT-PCR Kit	Applied Biosystems	Cat# 4368813
Experimental Models: Organisms/Strains		
D. melanogaster: y w	Stanewsky lab, University of Münster [4],	N/A
D. melanogaster: y w;;ss^{1} cry^{b}	Stanewsky lab; University of Münster [4],	N/A
D. melanogaster: y w;; cry^{b}	Rouyer lab, CNRS, Gif-sur-Yvette [28],	N/A
D. melanogaster: ninaE^{17}	Helfrich-Förster lab, University of Würzburg [53],	N/A
D. melanogaster: Rh5^{2}	Desplan lab, New York University [54],	N/A
D. melanogaster: Rh6^{1}	Desplan lab, New York University [55],	N/A
D. melanogaster: Gq^{1}	Hasan lab, National Centre for Biological Sciences, Bangalore [49],	N/A
D. melanogaster: Plc21C^{P319}	Hasan lab, National Centre for Biological Sciences, Bangalore [40],	N/A
Oligonucleotides		
rp49 sense 5′-CGATATGCTAAGCTGTCGCACA-3′	[56]	N/A
rp49 antisense 5′-CGCTTGTTCGATCCGTAACC-3′	[56]	N/A
PLC21C sense 5′-CCGCTTTTGGGGTTCTCTCT-3′	Stanewsky lab, University of Münster, this paper	N/A
PLC21C antisense 5′-TCTGGTCGACCCAGTAG AGG-3′	Stanewsky lab, University of Münster, this paper	N/A
Software and Algorithms		
MATLAB	Mathworks, Inc	Version 2014a
Flytoolbox	Levine Lab, University of Toronto [57],	stanewsky@uni-muenster.de
FIJI	ImageJ	https://imagej.nih.gov/ij/
GIMP	GNU Image Manipulation Program	https://www.gimp.org/
LED controller	[58]	https://github.com/PolygonalTree/Led-control
pClamp	Molecular Devices	Version 10

CONTACT FOR REAGENT AND RESOURCE SHARING

Further information and requests for resources and reagents should be directed to and will be fulfilled by the Lead Contact, Ralf Stanewsky (stanewsky@uni-muenster.de)

EXPERIMENTAL MODEL AND SUBJECT DETAILS

Flies

Flies were raised with a 12-h:12-h LD cycle on standard *Drosophila* medium (0.7% agar, 1.0% soy flour, 8.0% polenta/maize, 1.8% yeast, 8.0% malt extract, 4.0% molasses, 0.8% propionic acid, 2.3% nipagen) at 25°C and were collected ~3–5 d post eclosion.

y w flies, as well as *norpA*^{P41} and *cry*^{b} flies have been described previously [4]. The *Rh5*^{2} and *Rh6*^{1} alleles harbor intragenic deletions of coding sequences [54, 55], while *Gq*^{1} is a strong hypomorph [49]. The loss-of-function allele *ninaE*^{17} removes large parts (1.6 kb) of the Rh1 DNA coding region [53]. The hypomorphic allele *Plc21C*^{P319} harbors a *P*-element insertion in the first intron of the *Plc21C* gene resulting in blunted antennal olfactory responses and reduced *Plc21C* mRNA levels [40].

METHOD DETAILS

Behavior analysis

3 to 5 day old male flies were individually recorded using the *Drosophila* Activity Monitor System (DAM2; Trikinetics) in tubes containing food consisting of 2% agar and 4% sucrose. The monitors were located inside a temperature-controlled incubator, where cold white LED strips (5050 surface-mount device cool white LED strips, 3000-4000 K, Figure S1B) were attached. The LEDs were controlled using an Arduino with a custom made shield, enabling the Arduino to switch the lights on and off in conjunction with an open source desktop app [58]. As shown in Figure S1 lights were ramped for 2 hours from 0 to 60 μWatt/cm^2 (~180 lux) at the beginning of the day and in the opposite direction in the evening. For the first 4 days, the flies were kept at the same regime that they were raised in, with the addition of a temperature cycle of 25°C during ‘lights on’ and 16°C during ‘lights off’. On the last day before the change, the temperature was kept constant at 25°C. On that day, the night was prolonged by 6 hours. The flies were in the new regime for 7 days, after which they were released into DD for another 5 days. The actograms and histograms were generated in MATLAB using the flytoolbox [57]. The histograms of the second LD phase were done from the data of the last 3 days, to avoid any transients. The quantification of the phase was done as in [28], where in a custom made Excel macro, individual histograms for each day were assessed to determine the evening activity peak. The averages, SEM and plotting of the results were done in R.

Electroretinogram recordings

Electroretinograms (ERG) were recorded as described previously (e.g [59]) from young (2-5 days old) dark-reared flies of either sex immobilized with low melting point wax in truncated pipette tips. Recordings were made with low resistance (~10 MΩ) glass microelectrodes filled with fly Ringer (140 mM NaCl, 5 mM KCl, 1.5 mM $CaCl_2$, 4 mM $MgCl_2$) ‘punched’ through the cornea into the retina, with a similar electrode inserted into the head capsule near the ocelli as reference. Light stimulation came from a “warm white” power LED (Cairn Research, Figure S1C) delivered to within ~5 mm of the eye via a liquid-filled light guide (diameter 5 mm). Intensity of the stimulus was approximately 10^7 effectively absorbed photons per second per photoreceptor. All flies were on a white-eyed (*w*^{1118}) background.

Immunohistochemistry

To study PER levels, immunohistochemical analysis were performed as previously described [60]. Briefly, prior to collection, young adult males were entrained for 3 days under the same 12:12 hr LD conditions as were used for the behavior experiments. At the determined time points the flies were fixed in 4% PFA for 4h at 4°C. After fixation, the samples were washed 6 times with 0.1 M phosphate buffer (pH 7.4) with 0.1% Triton X-100 (PBS-T) at room temperature (RT). Brains were dissected in PBS, were then blocked with 10% goat serum in 0.5% PBS-T for 2 hr at RT and stained with pre-absorbed rabbit anti-PER (1:1000) [52] and monoclonal mouse anti-PDF C7 (DSHB, 1:200) in 10% goat serum in 0.5% PBST for at least 48h at 4°C. After washing 3 times by PBS-T, the samples were incubated at 4°C overnight with goat anti-rabbit AlexaFluor 488 nm (1:250) and goat anti mouse AlexaFluor 647 nm (Molecular Probes) in PBS-T. Brains were washed 3 times in PBS-T before being mounted in Vectashield. The images were taken using a Leica SP8 confocal microscope, keeping the same conditions for each experiment. Clock neurons were identified either directly (s-LNv and l-LNv) via expression of the neuropeptide PDF, or by their position within the brain with respect to the PDF+ LNv cell bodies and PDF processes projecting from the s-LNv into the dorsal protocerebrum (for 5th-LNv, LNd, DN1, and DN2). For time course quantification, pixel intensity of mean and background staining in each neuronal group was measured by FIJI [61]. For each cell three measurements were taken, as well as 3 measurements of the background for the corresponding slice. The data were analyzed and represented using R. After background subtraction, the measurements were normalized to the values of each of the neuronal groups obtained for *y w* at ZT22. The images shown in Figure 3 A and B were processed with GIMP.

RNA isolation and RT-PCR

To extract the RNA, 20-50 flies of the indicated genotype were collected in 2 mL RNAlater (Ambion) supplemented with 100 μl 0.1% PBST to improve RNAlater penetration. Flies were incubated overnight at 4°C, before 30 retinas and 20 brains were dissected in cold RNAlater. Total RNA was extracted using the RNeasy kit (QIAGEN) following the manufacturer’s instructions. The eluted RNA was immediately used for cDNA synthesis using the Reverse Transcription Reagents Kit (Applied Biosystems) using 1 μg of total RNA as a template. The cDNA was diluted and amplified via PCR with the following oligonucleotides: rp49 sense 5′-CGATATGCTAAGCTGTCGCACA-3′, rp49 antisense 5′-CGCTTGTTCGATCCGTAACC-3′ (as in [56]), PLC21C sense 5′-CCGCTTTTGGGGTTCTCTCT-3′ and PLC21C antisense 5′-TCTGGTCGACCCAGTAGAGG-3′. Bands were separated on 2% agarose gels and their intensities measured using ImageJ FIJI plugin and normalized against the intensity value of rp49.

QUANTIFICATION AND STATISTICAL ANALYSIS

Statistical significance was calculated in R by one-way ANOVA followed by Tukey's test for post hoc analysis (Figures 2E, 3C–E, 5D, S2), or by Dunnet's multiple comparison (Figures 4C, 4D, 5F).

DATA AND SOFTWARE AVAILABILITY

Raw data and locomotor activity analysis code will be provided upon request by Lead Contact, Ralf Stanewsky (stanewsky@uni-muenster.de)

Current Biology, Volume *28*

Supplemental Information

Non-canonical Phototransduction Mediates Synchronization of the *Drosophila melanogaster* Circadian Clock and Retinal Light Responses

Maite Ogueta, Roger C. Hardie, and Ralf Stanewsky

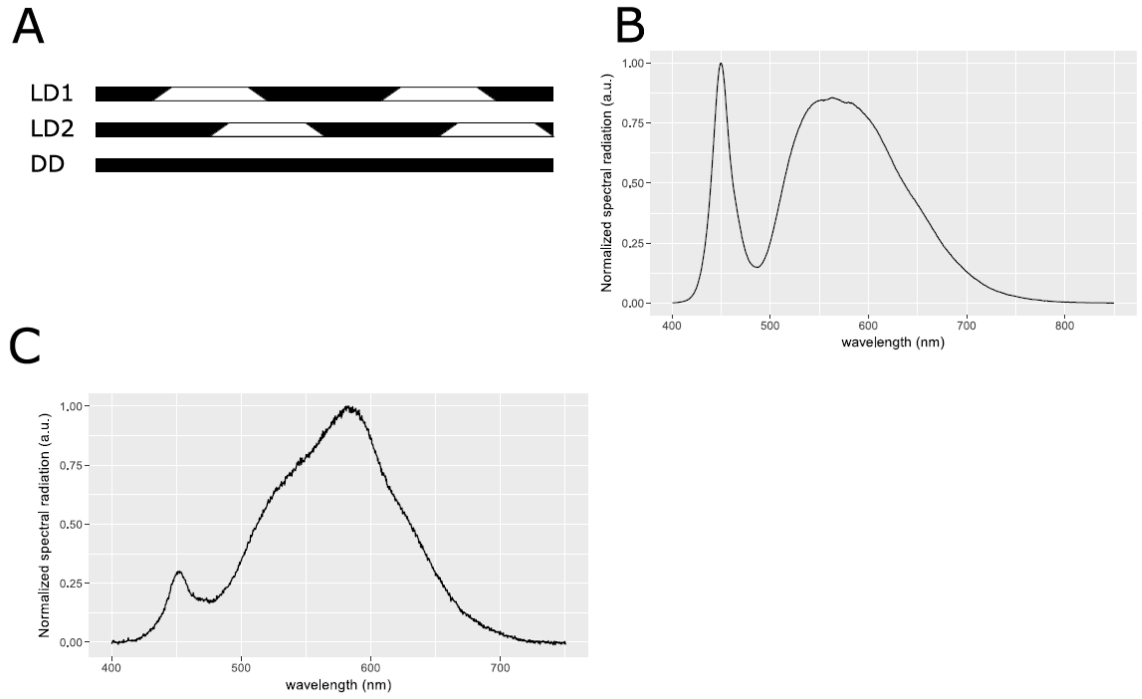

Figure S1. Characteristics of light conditions used in the experiments. Related to Figures 1, 2, 4, 5. (A) Light profile during the behavioural tests. During both LD periods, the lights are ramped for two hours simulating dawn and dusk respectively. The phase of LD1 is the same one as where the flies where reared, while LD2 has a phase shift of 6 hours. (B) Spectrum of the cold white LEDs used in the experiment. (C) Spectrum of the light source used for ERG recordings.

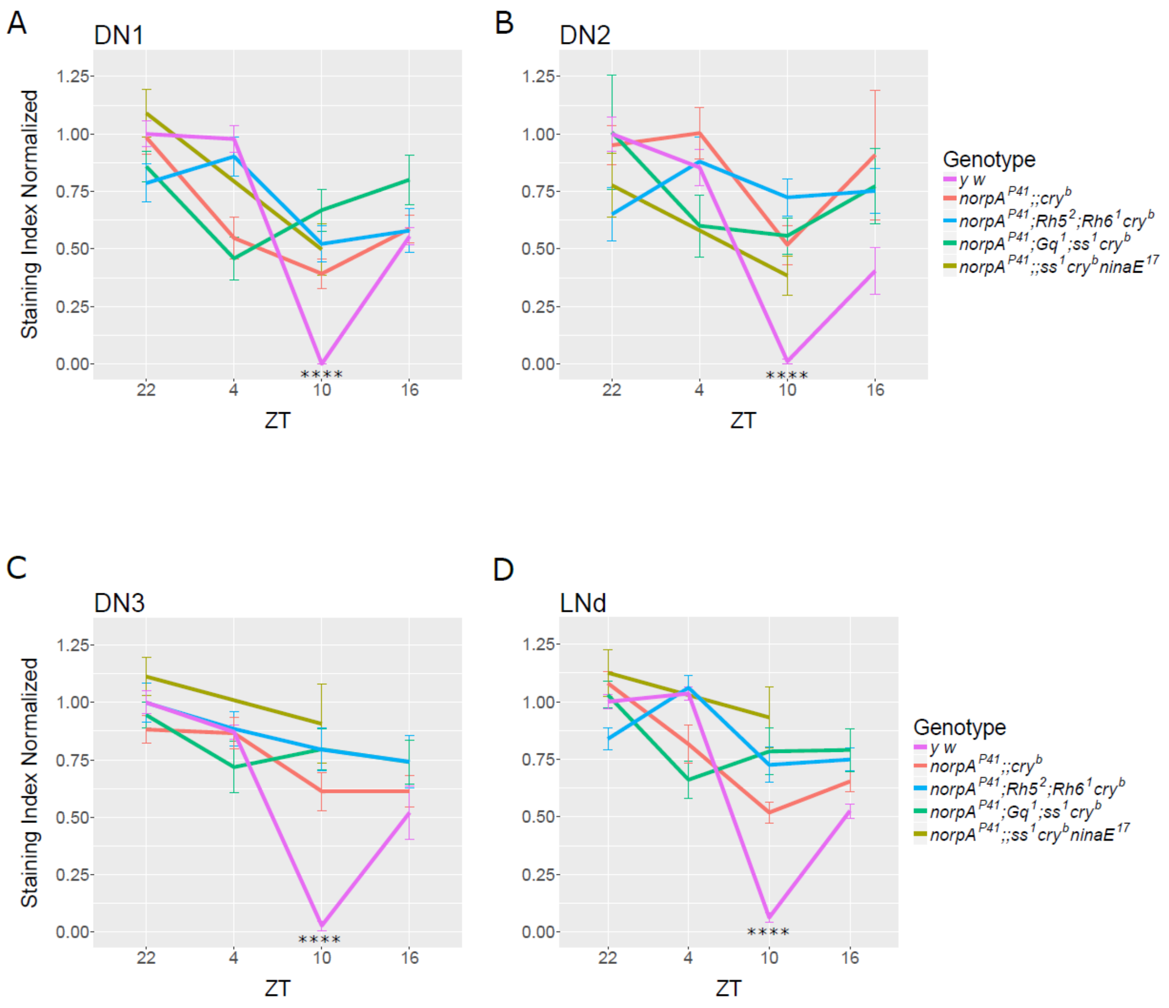

Figure S2. PER does not oscillate in the DNs and LNds of *norpA*^{P41}*;;cry*^{b} flies. Related to Figure 3. Quantification of PER expression in DN1 (A), DN2 (B), DN3 (C) and LNd (D) of the different genotypes, normalized to the values of *y w* at ZT22.Only *y w* flies show a trough of PER expression at ZT10 for all neuronal groups. Error bars represent SEM (n numbers in Table S1). **** represents p<0.0000001.

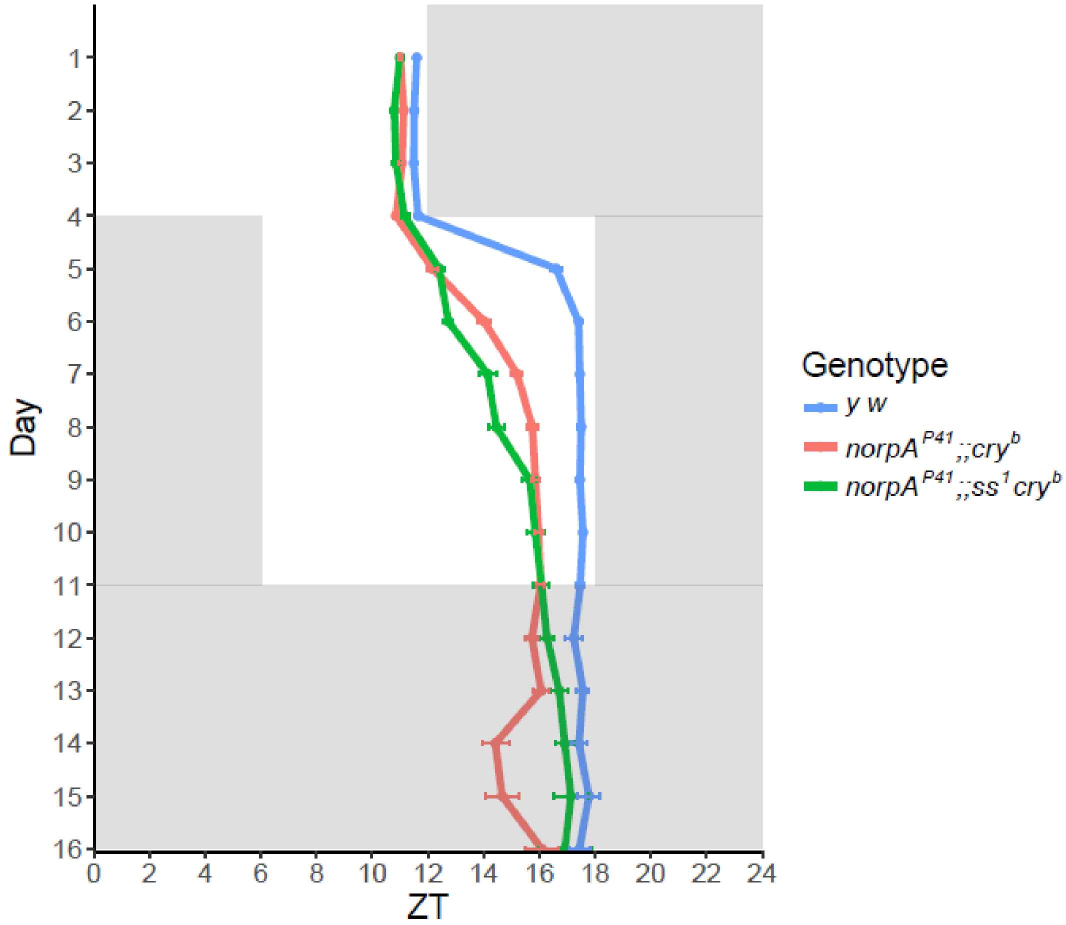

Figure S3. The hypomorphic *ss*[1] allele does not further compromise resynchronisation of *norpA*[P41] *cry*[b] flies. Related to Figure 2. Flies of the indicated genotypes were tested for resynchronisation to a 6 hr delayed LD cycle as in Figures 1, 2, and 5. Because loss-of-function alleles of *ss* reduce expression of Rh6 in R8 cells [S1], we tested if *norpA*[P41] *ss*[1] *cry*[b] flies needed longer for resynchronisation compared to *norpA*[P41] *cry*[b] flies. There was no significant differences between both genotypes, demonstrating that the lack of resynchronisation in *norpA*[P41] *ninaE*[17] *ss*[1] *cry*[b] flies (Figure 2C) is caused by the absence of Rh1 and not by reduction in Rh6 (see Discussion for details). n: *y w* 58, *norpA*[P41] *cry*[b] 40, *norpA*[P41] *ss*[1] *cry*[b] 21.

Table S1. Number of hemispheres quantified for PER expression. Related to Figure 2 and S2.

Genotype	ZT	Hemispheres
y w	22	52
	4	8
	10	37
	16	8
norpA^{P41};;cry^{b}	22	51
	4	19
	10	24
	16	17
norpA^{P41};Rh5^{2};Rh6^{1} cry^{b}	22	18
	4	7
	10	23
	16	11
norpA^{P41};Gq^{1}; ss^{1}cry^{b}	22	35
	4	12
	10	21
	16	6
norpA^{P41};;ninaE^{17} ss^{1} cry^{b}	22	13
	10	12

Numbers correspond to Zeitgeber Time (ZT) during a 12 hr : 12 hr LD cycle at 25°C (lights on at ZT0, lights off at ZT12) and to the number of hemispheres that were quantified at a given ZT for each genotype. Brains were sampled from 2 to 5 independent experiments.

Supplemental References

S1. Wernet, M.F., Mazzoni, E.O., Çelik, A., Duncan, D.M., Duncan, I., and Desplan, C. (2006). Stochastic spineless expression creates the retinal mosaic for colour vision. Nature *440*, 174–180.
